# Oregano Essential Oil Alleviates Diquat-Induced Oxidative Stress in Calves and Modulates Rumen Microbiota

**DOI:** 10.3390/antiox15101276

**Published:** 2026-10-02

**Authors:** Meixian Zhang, Huan Chen, Ting Liu, Yunbo Wang, Yining Cheng, Yingquan Hao

**Affiliations:** 1 College of Animal Science and Technology, Gansu Agricultural University, No. 1 Yingmen Village Anning, Lanzhou 730070, China; zmx15719616201@163.com (M.Z.); 17723395157@139.com (H.C.); 18093771432@163.com (Y.W.); haoyq17@163.com (Y.H.); 2 Gansu Key Laboratory of Herbiorous Animal Biotechnology, Gansu Agricultural University, No. 1 Yingmen Village Anning, Lanzhou 730070, China; 3 College of Veterinary Medicine, Gansu Agricultural University, No. 1 Yingmen Village Anning, Lanzhou 730070, China; yncheng5@163.com

**Keywords:** diquat, Nrf2-related antioxidant genes, oregano essential oil, oxidative stress, rumen metabolome, rumen microbiota

## Abstract

Oxidative stress can impair calf health and performance, and oregano essential oil (OEO) has potential antioxidant activity. This study evaluated dietary OEO in a diquat-induced oxidative-stress model using 24 weaned male Holstein calves assigned to a 2 × 2 factorial design with OEO supplementation (0 or 8 g/day) and diquat challenge (saline or 8 mg/kg body weight). Growth performance, serum biochemical, antioxidant, oxidative-damage and inflammatory indices, hepatic Nrf2-related antioxidant gene expression, rumen microbiota, and rumen metabolomics were assessed. The results showed that growth responses were phase dependent: OEO increased overall average daily gain (ADG) and dry matter intake (DMI), whereas diquat reduced overall ADG; no OEO × diquat interaction was detected for overall growth outcomes. The OEO group had higher serum SOD, GSH-Px, and T-AOC levels and lower levels of selected oxidative-damage and inflammatory indices, although the responses varied by endpoint and time point. Hepatic RT-qPCR showed treatment-associated differences in Keap1, HMOX1, GCLC, GCLM, and SOD1 expression. Rumen 16S rRNA gene sequencing showed treatment-associated shifts in microbial community composition, whereas PICRUSt2 predicted differences in microbial functional potential, including amino-acid-metabolism-related functions. Untargeted metabolomics identified treatment-associated differences in rumen metabolite profiles and KEGG pathway-enrichment patterns. Correlation analysis indicated that OEO-associated characteristic metabolites were positively correlated with antioxidant markers and negatively correlated with oxidative-damage and pro-inflammatory indices. Overall, OEO supplementation was associated with improved antioxidant status and distinct rumen microbial-metabolic profiles in diquat-challenged calves, supporting its potential as a natural feed additive, while the involvement of specific molecular pathways requires further validation.

## 1. Introduction

In intensive rearing systems, the weaning period is regarded as the most critical and challenging phase for calves [1]. During this stage, calves not only undergo a dietary transition from liquid milk to solid feed but must also adapt to group transfers, increased stocking densities, and heightened pathogen pressure. These changes collectively pose significant challenges to the calves’ physiological homeostasis [2]. Multiple stressors can readily disrupt the calf’s redox balance, inducing excessive ROS production and consequently triggering oxidative stress. The detrimental effects of oxidative stress on calves are pronounced: research indicates it impairs lymphocyte function in newborn calves, thereby weakening immunity [3]; it degrades crucial biomolecules such as proteins, lipids, and DNA [4,5]; and it further compromises the body’s antioxidant defense systems, diminishing production performance [6]. When the body sustains oxidative stress damage, the liver—as the central organ for biosynthesis, metabolism, and defense—is particularly vulnerable to ROS assault [7]. Research indicates that under oxidative stress, calf liver antioxidant enzyme activity significantly declines while levels of lipid peroxidation products like MDA rise, leading to increased cell membrane permeability, organelle dysfunction, and even apoptosis [8]. Consequently, measures to alleviate oxidative stress in calves are imperative.

Diquat is a commonly used chemical agent for modeling oxidative stress. It sustainably generates ROS and specifically damages the liver, having been extensively employed in oxidative stress studies involving piglets and poultry [9,10,11]. As a key transcription factor regulating the antioxidant response, Nrf2 recognizes and binds to antioxidant response elements (AREs), thereby upregulating the expression of multiple antioxidant enzymes and enhancing cellular defense capabilities [12,13,14]. Under normal conditions, Nrf2 interacts with Kelch-like ECH-associated protein 1 (Keap1) in the cytoplasm, leading to Nrf2 degradation via the ubiquitin-proteasome pathway and preventing its nuclear accumulation [14,15]. Under oxidative stress, signals from reactive oxygen species or electrophiles dissociate Nrf2 from Keap1, allowing Nrf2 to enter the nucleus and initiate transcription of downstream antioxidant genes [16]. Consequently, regulation of Nrf2-related antioxidant responses is recognized as an important component of cellular defence against oxidative stress.

In recent years, plant extracts have gained significant attention due to their rich content of antioxidant active components. Phenolic compounds, known for their strong antioxidant properties, can scavenge free radicals and chelate metals, sometimes surpassing the effectiveness of vitamins E and C [17,18]. OEO, a typical plant extract rich in carvacrol and thymol, has been shown to directly scavenge ROS, reduce MDA levels, promote the synthesis of SOD and GSH-Px, and inhibit the Nuclear Factor kappa-light-chain-enhancer of activated B cells (NF-κB) pathway to reduce the expression of inflammatory factors [19,20,21]. Additionally, our previous research found that OEO can promote growth and development in calves [22,23]. However, whether OEO-associated protection against diquat-induced oxidative stress in calves is accompanied by changes in Nrf2-related antioxidant gene expression and rumen microbial and metabolic profiles remains unclear.

Based on the above research background, this trial induced a stable oxidative stress state in weaned calves by intraperitoneal injection of diquat, with dietary supplementation of OEO. The aim was to evaluate whether dietary OEO pre-supplementation could modify the response of calves to a subsequent diquat-induced oxidative challenge, with emphasis on growth performance, serum antioxidant and inflammatory indices, hepatic Nrf2-related antioxidant gene expression, rumen microbial composition, and rumen metabolite profiles.

## 2. Materials and Methods

### 2.1. Ethical Statement

All experimental procedures were conducted in accordance with the Guidelines for the Care and Use of Laboratory Animals issued by the Ministry of Science and Technology of the People’s Republic of China. This protocol was reviewed and approved by the Animal Care and Use Committee of Gansu Agricultural University (Approval No.: GSAU-ETH-AST-2023-036).

### 2.2. Animals and Experimental Design

**Figure 1:**
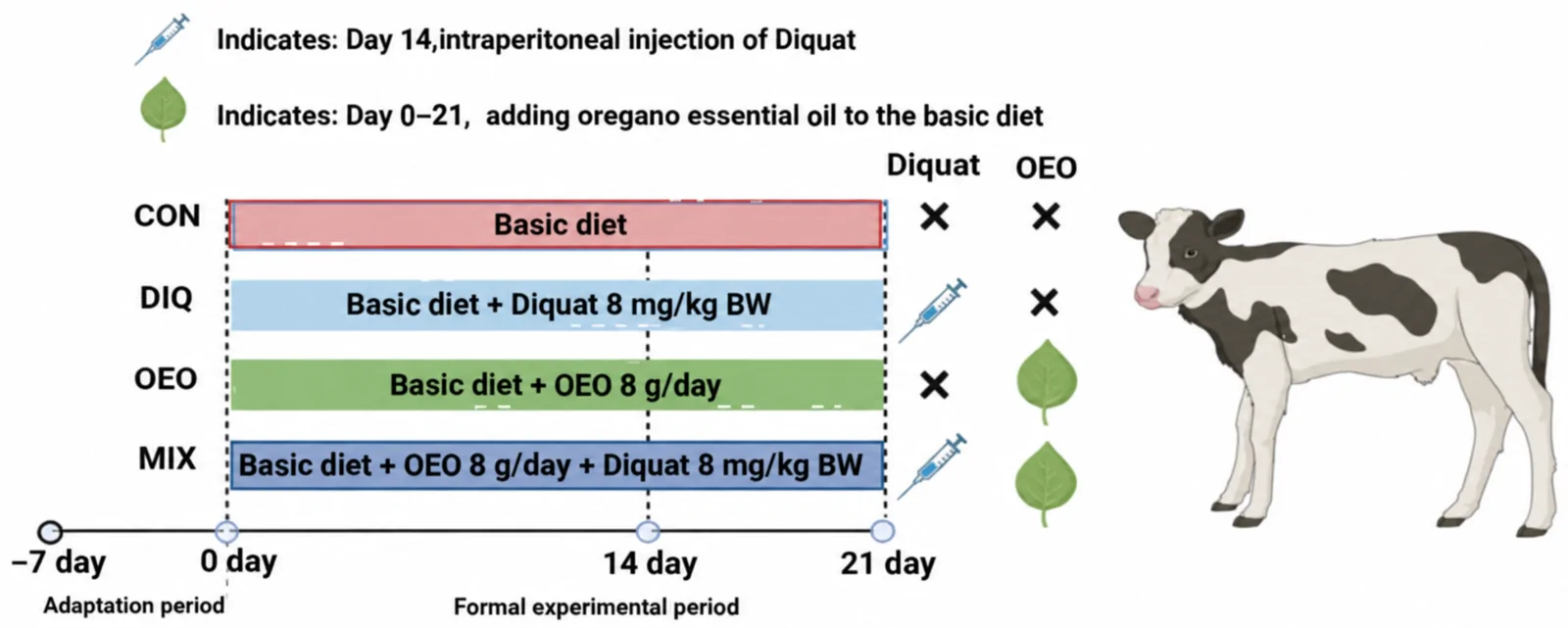
Schematic diagram of animal grouping and experimental treatment protocol.

The trial was conducted from July to August 2024 at the Liao Yuan Sheng Yuan Dairy Farm in Linxia City, Gansu Province, China. Twenty-four weaned male Holstein calves (90 ± 10 days; 115.00 ± 5.29 kg body weight) were randomly assigned to four groups (*n* = 6 per group) using a randomized block design (Figure 1): (1) Control group (CON), (2) Diquat group (DIQ), (3) OEO group (OEO), and (4) OEO and Diquat mixed group (MIX). Calves in the CON and DIQ groups were fed a basal diet, while those in the OEO and MIX groups received the basal diet manually mixed with OEO at a daily dose of 8 g/day. On day 14 of the trial, calves in the DIQ and MIX groups received an intraperitoneal injection of diquat (8 mg/kg BW, dissolved in 20 mL saline), while CON and OEO calves received an equal volume of saline (Figure 1). The trial period comprised a 7-day adaptation phase followed by a 21-day formal trial. The experimental design was intended to evaluate the protective effect of dietary OEO pre-supplementation against a subsequent oxidative challenge rather than the therapeutic reversal of established oxidative injury. Calves assigned to the OEO-containing groups received OEO for 14 days before diquat administration and continued to receive OEO thereafter. The diquat dose of 8 mg/kg BW was selected based on our previous calf study [24,25]. The OEO product was purchased from Ralco Nutrition Company (Marshall, MA, USA) and contained carvacrol (51.78%) and thymol (6.93%) as the main active components, with calcium carbonate, silica carbonate, and diatomaceous earth used as carriers, as previously reported by our group [24]. Diquat (purity ≥ 95%, C_12_H_12_Br_2_N_2_·H_2_O) was purchased from Sigma-Aldrich (St. Louis, MO, USA). Calves were housed individually in pens (1.5 × 2.0 m) fitted with sand bedding, which was cleaned daily and replaced every three days. Pens were disinfected weekly using a 0.2% sodium hypochlorite solution(Gansu Shenghuawei Co., Ltd., Lanzhou, China). All calves were maintained under the same management conditions throughout the experiment, including identical basal diet formulation, feeding schedule, and water supply. Feed ingredients and drinking water sources remained consistent among all treatment groups to minimize environmental variation. Animals had ad libitum access to water throughout the trial and were fed once daily at 09:00 h. The total mixed ration (TMR) comprised a pelleted(Gansu Dehua Feed Co., Ltd., Zhangye, China) grain mixture and oat hay(Gansu Dehua Feed Co., Ltd., Zhangye, China) in a 9:1 ratio (dry matter basis). The same batch of feed ingredients was used throughout the experimental period to ensure consistency of dietary composition among treatments. The composition and nutritional content of the basal diet are detailed in Table 1, conforming to the Chinese Dairy Cattle Feeding Standards (NY/T 34-2024) [26]. The calf barn provided ample lighting and good ventilation, meeting the Chinese Animal Husbandry Hygiene Standards (GB 13078-2017) [27].

### 2.3. Sample Collection

Daily records of feed offered and feed refused were maintained to calculate dry matter intake (DMI). Body weight (BW) was measured on days 1, 15, and 21 of the trial, and average daily gain (ADG) was calculated. Day 1 was used as the pre-diquat measurement, whereas days 15 and 21 corresponded to approximately 1 and 7 days after diquat challenge, respectively, allowing assessment of early and later post-challenge responses. Blood samples (10 mL) were collected at these same time points via jugular vein puncture using vacuum serum tubes (Becton, Dickinson and Company, Franklin Lakes, NJ, USA) at 9:00 a.m. prior to morning feeding. The blood samples were allowed to stand for 30 min and then centrifuged at 3500× *g* for 15 min at 25 °C to obtain serum. The serum samples were stored at −80 °C and subsequently sent to Beijing Huaying Biotechnology Research Institute (Beijing, China) for biochemical analysis. On day 21, at 2 h after morning feeding (11:00 a.m.), rumen fluid (30 mL, in 5 tubes) was collected from each calf via esophageal aspiration using a stomach tube. To prevent salivary contamination, the first 20 mL of rumen fluid was discarded. The rumen fluid samples were filtered through four layers of sterile gauze, immediately immersed and stored in liquid nitrogen, and transported to the laboratory under liquid-nitrogen conditions for 16S rRNA gene sequencing and metabolomic analysis. On day 21, following rumen fluid collection, three calves were randomly selected from each experimental group (*n* = 3 per group). The animals were mechanically stunned and immediately exsanguinated for euthanasia. Liver tissue samples of approximately 2 cm × 2 cm were rapidly excised from the same anatomical site (the right lobe) across all animals to ensure sampling consistency. The samples were immediately rinsed with sterile physiological saline (Shanghai Meiji Bio-Pharmaceutical Technology Co., Ltd., Shanghai, China) to remove adherent blood and debris. Each cleaned tissue sample was immediately snap-frozen and stored in liquid nitrogen until RNA extraction. All procedures for tissue collection and handling were performed on ice to minimize RNA degradation. The collected liver tissues were used for real-time quantitative PCR (qPCR) analysis to evaluate the expression of target genes.

### 2.4. Blood Analysis

Serum biochemical parameters were determined by Beijing Huaying Biotechnology Research Institute (Beijing, China). Serum superoxide dismutase (SOD, kit No. HY-M0001), glutathione peroxidase (GSH-Px, HY-M0004), total antioxidant capacity (T-AOC, HY-M0011), and catalase (CAT, HY-M0018) were measured using commercial colorimetric kits (Beijing Huaying Biotechnology Research Institute, Beijing, China) according to the manufacturer’s instructions, with the xanthine–xanthine oxidase method for SOD, the DTNB method for GSH-Px, the ABTS method for T-AOC, and the ammonium molybdate method for CAT. Serum 8-iso-prostaglandin F2α (8-iso-PGF2α, HY-NE144), interleukin-1β (IL-1β, HY-H0001), interleukin-6 (IL-6, HY-H0007), interleukin-10 (IL-10, HY-H0009), and tumor necrosis factor-α (TNF-α, HY-H0019) were quantified using competitive ELISA kits from the same manufacturer. Serum aspartate aminotransferase (AST), alanine aminotransferase (ALT), and alkaline phosphatase (ALP) activities were determined using kinetic assay kits (Zhongsheng Beikong Bio-Technology and Science Inc., Beijing, China). Serum malondialdehyde (MDA) was determined by the thiobarbituric acid (TBA) colorimetric method using a commercial kit (catalog number HY-M0003) provided by Beijing Huaying Biotechnology Research Institute (Beijing, China), following the manufacturer’s instructions.

### 2.5. RNA Extraction and RT-qPCR

Total RNA was extracted from liver tissue using TRIzol reagent (Thermo Fisher Scientific, Shanghai, China) according to the manufacturer’s instructions. RNA concentration and purity were determined using a NanoDrop ND-1000 spectrophotometer (Thermo Fisher Scientific, Shanghai, China). For each sample, 2 μg of total RNA was reverse-transcribed into cDNA using a reverse-transcription kit (Vazyme Biotech Co., Ltd., Nanjing, China) according to the manufacturer’s instructions.

Quantitative real-time PCR (RT-qPCR) was performed using a CFX Connect Real-Time PCR Detection System (Bio-Rad Laboratories, Hercules, CA, USA) with SYBR Green Master Mix (Takara, Tokyo, Japan). Each 20 μL reaction contained 10 μL of SYBR Green Master Mix, 7.2 μL of nuclease-free water, 2 μL of cDNA, 0.4 μL of forward primer (10 μM), and 0.4 μL of reverse primer (10 μM), corresponding to a final concentration of 0.2 μM for each primer. The amplification conditions were as follows: initial denaturation at 95 °C for 30 s, followed by 40 cycles of 95 °C for 5 s and 60 °C for 34 s. Melting-curve analysis was subsequently performed to assess amplification specificity.

Three calves were randomly selected from each treatment group for RT-qPCR analysis (*n* = 3 biological replicates per group). Each biological sample was analyzed in six technical replicates, and the mean Ct value of the six technical replicates was used for subsequent calculations. GAPDH was used as the reference gene. Relative expression levels of *Keap1*, *HMOX1*, *NQO1*, *GPX4*, *GCLC, GCLM*, and *SOD1* were calculated using the 2^−ΔΔCt^ method, with the mean expression level of the CON group used as the calibrator. Primer sequences were designed by Sangon Biotech Co., Ltd. (Shanghai, China) using its online primer design tool (https://store.sangon.com/primerDesign, accessed on 16 January 2025). and the sequences are listed in Table 2.

### 2.6. 16S rRNA Gene Sequencing and Metabolomic Analysis

16S rRNA Sequencing: Microbial DNA was extracted from rumen fluid using the E.Z.N.A.^®^ Soil DNA Kit (Omega Bio-tek, Norcross, GA, USA). Using the extracted DNA as template, barcoded specific primers 338F (5′-ACTCCTACGGGAGGCAGCAG-3′) and 806R (5′-GGACTACHVGGGTWTCTAAT-3′) [28] were employed for PCR amplification of the 16S rRNA gene V3–V4 variable region. Reaction conditions: 4 μL 5× FastPfu buffer, 2 μL 2.5 mM dNTPs, 0.8 μL each of forward and reverse primers (5 μM), 0.4 μL FastPfu DNA polymerase, 10 ng template DNA, supplemented with ddH_2_O to 20 μL. Amplification programme: 95 °C pre-denaturation for 3 min; 27 cycles (95 °C for 30 s, 55 °C for 30 s, 72 °C for 45 s); final extension at 72 °C for 10 min, then stored at 4 °C. PCR products were detected via 2% agarose gel electrophoresis, purified using a PCR Clean-Up Kit (YuHua, Shanghai, China), and quantified with Qubit 4.0 fluorometer (Thermo Fisher Scientific, Waltham, MA, USA). Sequencing libraries were constructed using the NEXTFLEX^®^ Rapid DNA-Seq Kit and subjected to 2 × 250 bp paired-end sequencing on the Illumina NextSeq 2000 platform (Illumina, San Diego, CA, USA) at Shanghai Meiji Biotechnology Co., Ltd. (Shanghai, China).

Metabolite Extraction and LC-MS Analysis: For rumen metabolomics, 100 μL of rumen fluid was mixed with 400 μL of pre-chilled extraction solvent (acetonitrile:methanol = 1:1, containing 0.02 mg/mL L-2-chlorophenylalanine as internal standard). The mixture was vortexed for 30 s and subjected to low-temperature ultrasonic extraction (5 °C, 40 kHz) for 30 min. After incubation at −20 °C for 30 min, the extract was centrifuged (4 °C, 13,000 *g*, 15 min). The supernatant was dried under a nitrogen stream. The residue was reconstituted in 100 μL of solvent (acetonitrile:water = 1:1), vortexed, centrifuged, and the supernatant was transferred to an injection vial for LC-MS analysis. A quality control (QC) sample was prepared by pooling equal volumes of all samples. QC samples were analyzed intermittently throughout the batch to monitor instrument stability. Analysis was performed using a ultra-high-performance liquid chromatography-orbitrap mass spectrometry system (UHPLC-Orbitrap Exploris 480, Thermo Fisher Scientific, Waltham, MA, USA). Chromatographic separation employed an ACQUITY HSS T3 column (100 mm × 2.1 mm, 1.8 μm; Waters Corporation, Milford, MA, USA). Mobile phase A consisted of water:acetonitrile = 95:5 (containing 0.1% formic acid), while mobile phase B comprised acetonitrile:isopropanol:water = 47.5:47.5:5 (containing 0.1% formic acid). The gradient elution programme was as follows: 0–2 min, 2% B; 2–15 min, 2–100% B; 15–17 min, 100% B; 17–17.1 min, 100–2% B; 17.1–20 min, 2% B. Flow rate was 0.40 mL/min, column temperature was 40 °C, and injection volume was 3 μL. Mass spectrometry acquisition employed both positive and negative ion scanning modes, with a mass range of m/z 70–1050. Ion source parameters were as follows: positive ion spray voltage 3500 V, negative ion spray voltage –3000 V; sheath gas 50 arb, auxiliary gas 13 arb; ion transfer tube temperature 450 °C; collision energy set to a cyclic configuration of 20–40–60 V.

### 2.7. Data Processing

Growth-performance and blood data were analyzed using SAS 9.4 (SAS Institute Inc., Cary, NC, USA). The experiment was conducted as a randomized complete block design (RCBD) with a 2 × 2 factorial arrangement of treatments, including oregano essential oil supplementation (OEO; 0 or 8 g/day) and diquat challenge (saline or 8 mg/kg BW) as the two treatment factors. The 24 calves were ranked according to initial BW and stratified into six blocks of four calves with similar BW. Within each block, random numbers generated using the “RAND” function in Microsoft Excel were used to assign the four calves to the four treatment combinations, so that each block contained one calf from each of the CON, DIQ, OEO, and MIX groups, resulting in six calves per treatment. Block was included as a random effect.

Because diquat was administered on D14, measurements obtained on D1 were considered pre-diquat measurements. Although the calves had already been randomly assigned to their subsequent saline or diquat treatments at D1, any differences associated with future diquat assignment at that time were considered to reflect random between-animal variation rather than a biological effect of diquat. Therefore, D1 measurements were summarized descriptively, and no inferential tests were performed for OEO, future diquat assignment, or the OEO × diquat interaction. Because OEO supplementation had already begun before D1 measurements were obtained, D1 was considered a pre-diquat measurement rather than a strict pre-OEO baseline.

For BW and blood variables measured on D1, D15, and D21, the D15 and D21 outcomes were analyzed separately by analysis of covariance (ANCOVA) using the MIXED procedure. The corresponding D1 measurement was included as a covariate to account for inter-animal variation present before the diquat challenge. The model was:

*Y_ijkl_* = *μ* + *βX_ijk__l_* + *O_i_* + *D_j_* + (*O* × *D*)*_ij_* + *B_l_* + *ε_ijkl_*

where *Y_ijkl_* is the D15 or D21 response variable; *μ* is the overall mean; *X_ijk__l_* is the corresponding D1 covariate; *β* is the regression coefficient for the covariate; *O_i_* and *D_j_* are the fixed effects of OEO and diquat, respectively; (*O* × *D*)*_ij_* is the OEO × diquat interaction; *B_l_* is the random effect of block; and *ε_ijkl_* is the residual error.

For D15 and D21 outcomes, least-squares means (LSMeans) for the four treatment combinations (CON, DIQ, OEO, and MIX) were estimated at the overall mean of the corresponding D1 covariate. All six pairwise comparisons among the four treatment-combination LSMeans were adjusted using Tukey’s method to control the family-wise type I error rate. When the OEO × diquat interaction was significant, Tukey-adjusted pairwise comparisons among the treatment combinations were used to interpret the interaction. When the interaction was not significant, inference was based primarily on the main effects of OEO and diquat, with treatment-combination comparisons treated as secondary. Degrees of freedom for fixed-effect tests were adjusted using the Kenward–Roger (KR) method.

For growth-performance variables, only the effect of OEO was analyzed during the pre-diquat period (D1–14), and no inferential tests were conducted for future diquat assignment or the OEO × diquat interaction. Post-diquat (D15–21) and overall (D1–21) ADG and DMI were analyzed using the complete 2 × 2 factorial RCBD model. For D1–21 outcomes, effects attributed to diquat were interpreted as cumulative effects of the assigned treatment regimen because actual diquat exposure occurred only after D14.

Model assumptions were evaluated using model residuals. Residual normality was assessed using the Shapiro–Wilk test together with Q–Q plots, whereas homogeneity of variance was evaluated using Levene’s test and residual-versus-fitted plots. For ANCOVA models, homogeneity of regression slopes was additionally assessed by testing interactions between the D1 covariate and the treatment factors. Data are presented as least-squares means ± SEM, and differences were considered significant at *p* < 0.05.

All bioinformatics analyses were performed on the Meiji BioCloud platform (https://cloud.majorbio.com). Microbiome raw data underwent quality control using fastp (v0.19.6) [29], with sequence assembly performed by FLASH (v1.2.11) [30]. OTU clustering was conducted at 97% similarity using UPARSE (v7.1) [31], followed by chimerism removal. Sequences annotated as mitochondrial or chloroplast were excluded. To eliminate sequencing depth variation, all sample reads were downsampled to 20,000 reads, achieving a post-downsampling Good’s coverage index exceeding 99%. Species annotation was performed using RDP Classifier (v2.11) [32] against the Silva database (v138) with a confidence threshold of 70%. Alpha-diversity indices (Chao1, Shannon, Simpson, and ACE) were calculated using mothur [33]. Overall differences among the four treatment groups for each alpha-diversity index were assessed using the Kruskal–Wallis test. For taxa-level comparisons, *p* values were adjusted for multiple testing using the Benjamini–Hochberg false discovery rate (FDR) procedure, and FDR-adjusted *p* < 0.05 was considered statistically significant. Beta diversity was assessed using principal coordinate analysis (PCoA) based on Bray–Curtis dissimilarities. Overall differences in microbial community composition among the four treatment groups were evaluated using permutational multivariate analysis of variance (PERMANOVA). LEfSe [34] (LDA > 3, *p* < 0.05) identified species showing significant intergroup differences. Functional prediction analysis was conducted using PICRUSt2 [35]. For the predicted Level-2 functional profiles, intergroup comparisons were performed using one-way analysis of variance (ANOVA) followed by Tukey–Kramer post-hoc pairwise comparisons.

Metabolomic raw data were processed using Progenesis QI (Waters Corporation, Milford, MA, USA), including peak extraction, alignment, integration, and retention-time correction, generating a data matrix comprising retention times, mass-to-charge ratios, and peak intensities. Metabolite identification was performed by comparison with the HMDB, MetLin, and Majorbio proprietary databases. Data preprocessing was conducted on the Meiji BioCloud platform, including missing-value filtering using the 80% rule, imputation of remaining missing values with the minimum observed value, sum normalization, removal of QC features with RSD > 30%, and log10 transformation. Differential-metabolite screening was performed using an OPLS-DA model (R package ropls) in combination with univariate testing. For each pairwise comparison, two-tailed *t*-tests were performed, and the resulting *p* values were adjusted for multiple testing using the Benjamini–Hochberg false discovery rate (FDR) procedure. Candidate differential metabolites were screened using the predefined exploratory criteria of VIP > 1 and nominal *p* < 0.05, whereas the corresponding FDR-adjusted *p* values were used to provide a more conservative assessment of statistical evidence. Accordingly, metabolites meeting the VIP and nominal-P criteria were considered candidate differential metabolites rather than necessarily FDR-confirmed metabolites. The direction of change was determined according to the sign of the log2 fold change (log2FC); for each comparison, a positive log2FC for the first group relative to the second group indicated higher abundance, whereas a negative value indicated lower abundance. KEGG pathway annotation and enrichment analysis were performed using Fisher’s exact test. Associations between selected metabolites and host phenotypes were evaluated using Spearman’s rank correlation coefficient (r), and associations with |r| > 0.5 and nominal *p* < 0.05 were displayed in the correlation heatmaps. Because these metabolite–phenotype correlation analyses were exploratory, they were interpreted as associations rather than evidence of causation.

## 3. Results

### 3.1. Growth Performance

The effects of OEO supplementation and diquat challenge on growth performance are presented in Table 3. D1 BW was considered a pre-diquat measurement and is presented descriptively without inferential comparisons among treatments. OEO supplementation significantly affected BW on D15 (*p* < 0.05), whereas neither the diquat effect nor the OEO × diquat interaction was significant (*p* > 0.05). On D21, significant main effects of both OEO supplementation and diquat challenge were observed (*p* < 0.05), while the OEO × diquat interaction was not significant (*p* > 0.05).

During the pre-diquat period (D1–14), OEO supplementation significantly increased ADG (*p* < 0.05). During D15–21, ADG was significantly affected by both OEO supplementation and diquat challenge (*p* < 0.05), whereas their interaction was not significant (*p* > 0.05). The OEO group had greater ADG than the DIQ and MIX groups, and the CON group had greater ADG than the DIQ group (*p* < 0.05). Across the entire D1–21 period, significant main effects of OEO and diquat were detected for ADG (*p* < 0.05), with no significant interaction (*p* > 0.05). The OEO group had greater ADG than the CON, DIQ, and MIX groups, and the MIX group had greater ADG than the DIQ group (*p* < 0.05).

OEO supplementation also significantly increased DMI during D1–14 (*p* < 0.05). During D15–21, significant main effects of OEO and diquat were detected (*p* < 0.05), whereas the OEO × diquat interaction was not significant (*p* > 0.05). The OEO group had greater DMI than the DIQ group (*p* < 0.05), while the CON and MIX groups were intermediate. Across D1–21, OEO supplementation significantly affected DMI (*p* < 0.05), whereas neither the diquat effect nor the OEO × diquat interaction was significant (*p* > 0.05).

### 3.2. Blood Parameters

#### 3.2.1. Liver Function Indicators

The effects of OEO supplementation and diquat challenge on serum liver-function indices are presented in Table 4. On D15, significant main effects of both OEO and diquat were observed (*p* < 0.05), whereas the OEO × diquat interaction was not significant (*p* > 0.05). The DIQ group had greater AST activity than the CON, OEO, and MIX groups, and the MIX group had greater AST activity than the OEO group (*p* < 0.05). On D21, AST was again significantly affected by both OEO and diquat (*p* < 0.05), with no significant interaction (*p* > 0.05). The DIQ group had greater AST activity than the CON, OEO, and MIX groups, and the MIX group had greater AST activity than the OEO group (*p* < 0.05). For ALT, significant main effects of OEO and diquat were detected on D15 (*p* < 0.05), whereas the OEO × diquat interaction was not significant (*p* > 0.05). The DIQ and MIX groups had greater ALT activities than the CON and OEO groups (*p* < 0.05). On D21, OEO supplementation significantly affected ALT activity (*p* < 0.05), whereas neither the diquat effect nor the OEO × diquat interaction was significant (*p* > 0.05). The DIQ group had greater ALT activity than the OEO group (*p* < 0.05), while the CON and MIX groups were intermediate. For ALP, a significant main effect of diquat was detected on D15 (*p* < 0.05), whereas neither the OEO effect nor the OEO × diquat interaction was significant (*p* > 0.05).

#### 3.2.2. Antioxidant Indicators

Serum antioxidant and oxidative-stress indices are presented in Table 5. On D15, T-AOC was significantly affected by both OEO supplementation and diquat challenge (*p* < 0.05), whereas the OEO × diquat interaction was not significant (*p* > 0.05). The DIQ group had lower T-AOC than the CON, OEO, and MIX groups (*p* < 0.05). On D21, diquat significantly affected T-AOC (*p* < 0.05), whereas neither the OEO effect nor the OEO × diquat interaction was significant (*p* > 0.05). The CON group had greater T-AOC than the DIQ and MIX groups, while the OEO group was intermediate (*p* < 0.05).

CAT activity on D15 was significantly affected by OEO supplementation (*p* < 0.05), whereas neither the diquat effect nor the OEO × diquat interaction was significant (*p* > 0.05). The OEO and MIX groups had greater CAT activity than the CON group, while the DIQ group was intermediate (*p* < 0.05). On D21, a significant main effect of diquat was observed (*p* < 0.05), whereas neither OEO nor the OEO × diquat interaction was significant (*p* > 0.05).

For GSH-Px on D15, significant main effects of OEO supplementation and diquat challenge were detected (*p* < 0.05), whereas their interaction was not significant (*p* = 0.052). The OEO group had the highest GSH-Px activity, the DIQ group had the lowest, and the CON and MIX groups showed intermediate and similar values (*p* < 0.05). On D21, significant main effects of OEO and diquat, as well as a significant OEO × diquat interaction, were detected (all *p* < 0.001). The CON and OEO groups had higher GSH-Px activities than the MIX group, whereas the DIQ group had the lowest activity (*p* < 0.05).

For SOD, significant main effects of OEO and diquat were detected on D15 (*p* < 0.05), whereas their interaction was not significant (*p* > 0.05). The OEO and MIX groups had greater SOD activities than the CON group, while the DIQ group had the lowest activity (*p* < 0.05). On D21, a significant OEO × diquat interaction was detected (*p* < 0.05). The OEO group had the greatest SOD activity, the CON and MIX groups had intermediate and similar values, and the DIQ group had the lowest activity (*p* < 0.05).

For MDA on D15, OEO supplementation had a significant main effect (*p* < 0.05), whereas the main effect of diquat and the OEO × diquat interaction were not significant (*p* > 0.05). Tukey-adjusted pairwise comparisons showed that the DIQ group had a higher MDA concentration than the CON, OEO, and MIX groups, while no significant differences were detected among the latter three groups (*p* < 0.05). On D21, significant main effects of OEO supplementation and diquat challenge, as well as a significant OEO × diquat interaction, were detected (*p* < 0.05). The OEO group had a higher MDA concentration than the DIQ group, whereas the CON and MIX groups showed intermediate values (*p* < 0.05).

A significant OEO × diquat interaction was detected for 8-iso-PGF2α on D15 (*p* < 0.05). The DIQ group had the greatest concentration, the MIX group had an intermediate concentration, and the CON and OEO groups had lower and similar concentrations (*p* < 0.05). On D21, significant main effects of OEO and diquat were detected (*p* < 0.05), whereas their interaction was not significant (*p* > 0.05). The DIQ group had greater 8-iso-PGF2α concentrations than the other groups, and the MIX group had a greater concentration than the OEO group (*p* < 0.05), while the CON group was intermediate.

#### 3.2.3. Inflammatory Indicators

The effects of OEO supplementation and diquat challenge on serum inflammatory cytokines are presented in Table 6. On D15, a significant OEO × diquat interaction was detected for IL-1β (*p* < 0.05). The DIQ group had a greater IL-1β concentration than the CON, OEO, and MIX groups (*p* < 0.05), whereas no significant differences were observed among the latter three groups. On D21, diquat significantly affected IL-1β (*p* < 0.05), whereas neither the OEO effect nor the OEO × diquat interaction was significant (*p* > 0.05). The DIQ group had greater IL-1β concentrations than the CON and OEO groups, while the MIX group was intermediate (*p* < 0.05). A significant OEO × diquat interaction was also observed for IL-6 on D15 (*p* < 0.05). The DIQ group had a greater IL-6 concentration than the CON, OEO, and MIX groups (*p* < 0.05), whereas no significant differences were observed among the latter three groups. On D21, no significant effects of OEO, diquat, or their interaction were detected (*p* > 0.05). For IL-10, a significant OEO × diquat interaction was detected on D15 (*p* < 0.05). The OEO and MIX groups had greater IL-10 concentrations than the CON and DIQ groups, and the CON group had a greater IL-10 concentration than the DIQ group (*p* < 0.05). On D21, the OEO × diquat interaction remained significant (*p* < 0.05). The CON group had a greater IL-10 concentration than the OEO and DIQ groups, and the MIX group had a greater IL-10 concentration than the DIQ group (*p* < 0.05). For TNF-α on D15, a significant OEO × diquat interaction was detected (*p* < 0.05). The DIQ group had a greater TNF-α concentration than the CON, OEO, and MIX groups (*p* < 0.05), whereas no significant differences were observed among the latter three groups. On D21, no significant effects of OEO, diquat, or the OEO × diquat interaction were observed (*p* > 0.05).

### 3.3. RT-qPCR

**Figure 2:**
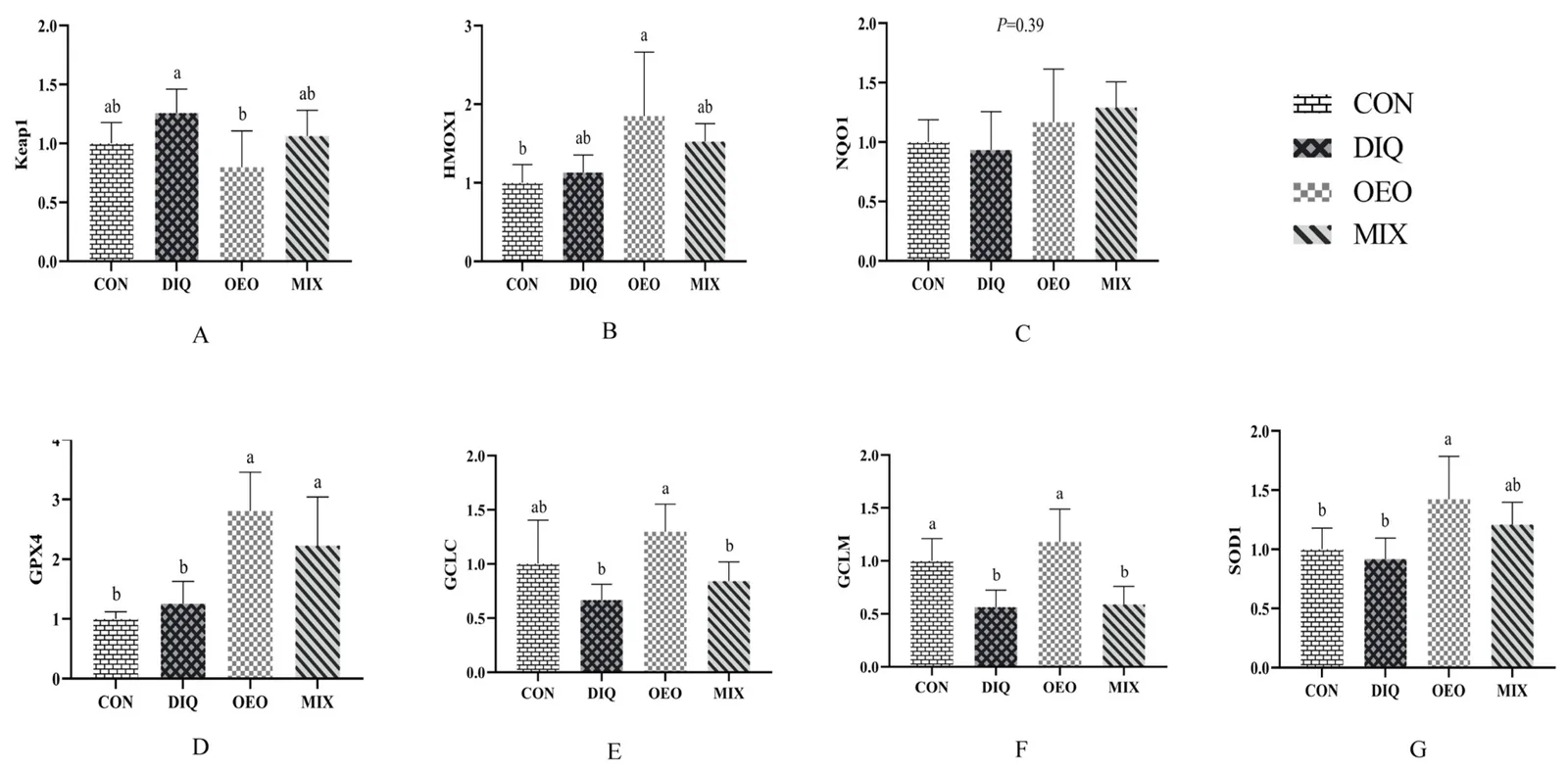
RT-qPCR of antioxidant genes in the liver. Note: (**A**) Keap1; (**B**) HMOX1; (**C**) NQO1; (**D**) GPX4, glutathione peroxidase 4; (**E**) GCLC; (**F**) GCLM; and (**G**) SOD1. The Y-axis represents relative mRNA expression (fold change vs. CON). Relative expression was calculated using the 2^−ΔΔCt^ method, normalized to *GAPDH*, and expressed as fold change relative to the CON group. Different lowercase letters indicate significant differences among groups (*p* < 0.05), whereas groups sharing the same letter are not significantly different.

As shown in Figure 2, RT-qPCR revealed treatment-associated changes in hepatic Nrf2-related antioxidant gene expression. *Keap1* expression was higher in the DIQ group than in the OEO group, whereas the CON and MIX groups showed intermediate values (*p* < 0.05). HMOX1 expression was higher in the OEO group than in the CON group, while the DIQ and MIX groups showed intermediate values (*p* < 0.05). No significant difference was detected in NQO1 expression among the groups (*p* = 0.39). GPX4 expression was higher in the OEO and MIX groups than in the CON and DIQ groups (*p* < 0.05). GCLC expression was higher in the OEO group than in the DIQ and MIX groups, whereas the CON group showed an intermediate value (*p* < 0.05). GCLM expression was higher in the CON and OEO groups than in the DIQ and MIX groups (*p* < 0.05). SOD1 expression was higher in the OEO group than in the CON and DIQ groups, while the MIX group showed an intermediate value (*p* < 0.05).

### 3.4. Rumen Microbiota

**Figure 3:**
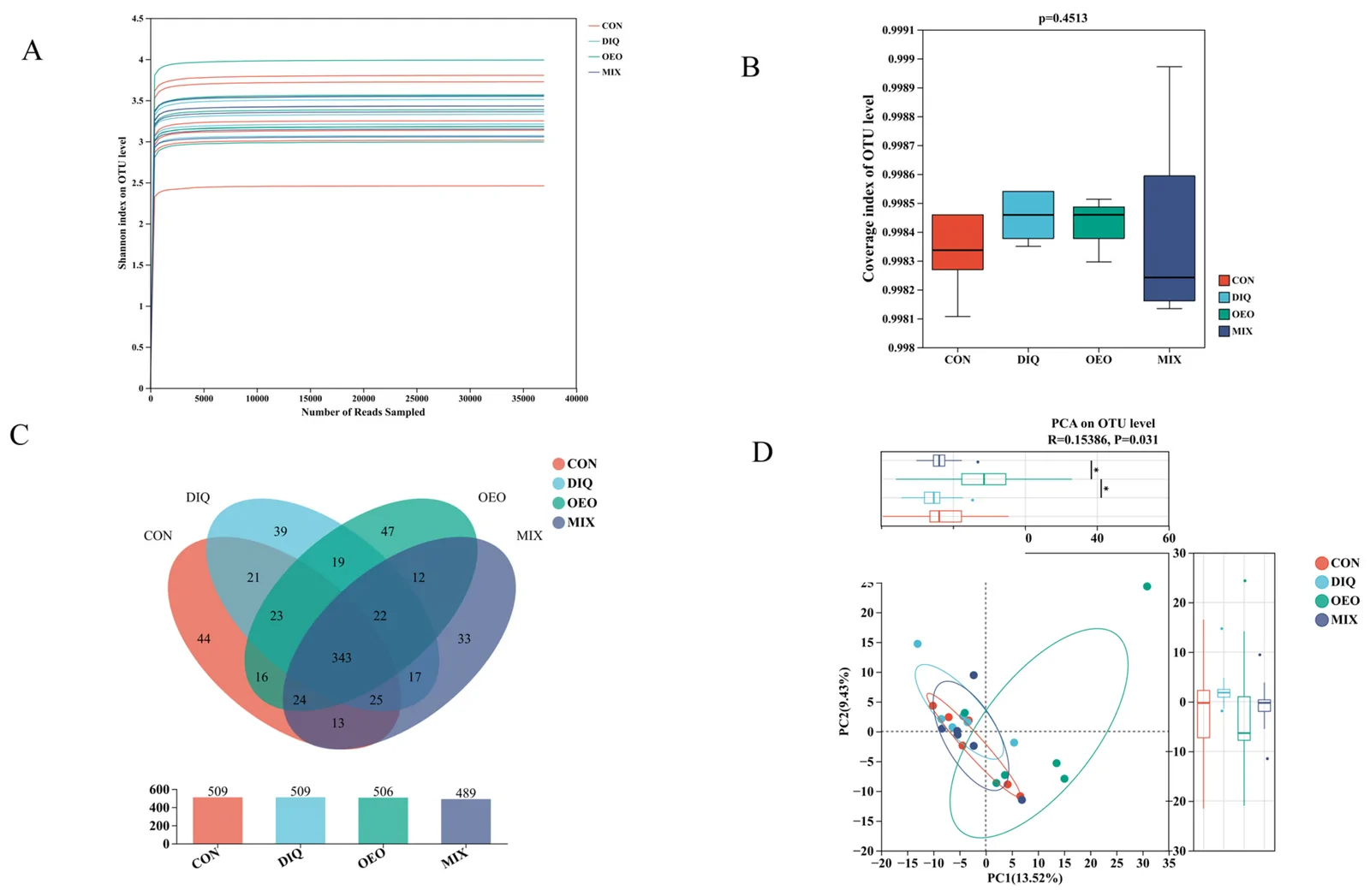
Effects of OEO and Diquat on the microbial community of calf rumen. Note: (**A**) Species Rarefaction curve; (**B**) Coverage index; (**C**) OTU Venn diagram; (**D**) PCoA based on Bray–Curtis distances; overall group differences were assessed by PERMANOVA. An asterisk (*) indicates a significant difference between groups (*p* < 0.05).

To investigate the effects of OEO and diquat on the rumen microbial communities of calves, we conducted 16S rRNA gene sequencing analysis. As shown in Figure 3A, the microbial diversity index curve transitioned from steep to flat, with all samples exhibiting Coverage indices exceeding 0.99 (Figure 3B). This indicates sequencing depth sufficiently captured the vast majority of microbial diversity within the samples. Venn diagram analysis revealed group-specific and shared operational taxonomic units (OTUs), with 44, 39, 47, and 33 unique OTUs identified in the CON, DIQ, OEO, and MIX groups, respectively, alongside 484 shared OTUs (Figure 3C). However, beta diversity analysis revealed differences in community composition. Principal coordinate analysis (PCoA) based on Bray–Curtis distances showed partial overlap among the four treatment groups, with a tendency toward separation of samples in the OEO group (Figure 3D). PERMANOVA detected a significant overall difference in microbial community composition among the groups (*p* = 0.031, R = 0.1539), with the low R value indicating a modest degree of separation.

#### 3.4.1. Alpha Diversity

**Figure 4:**
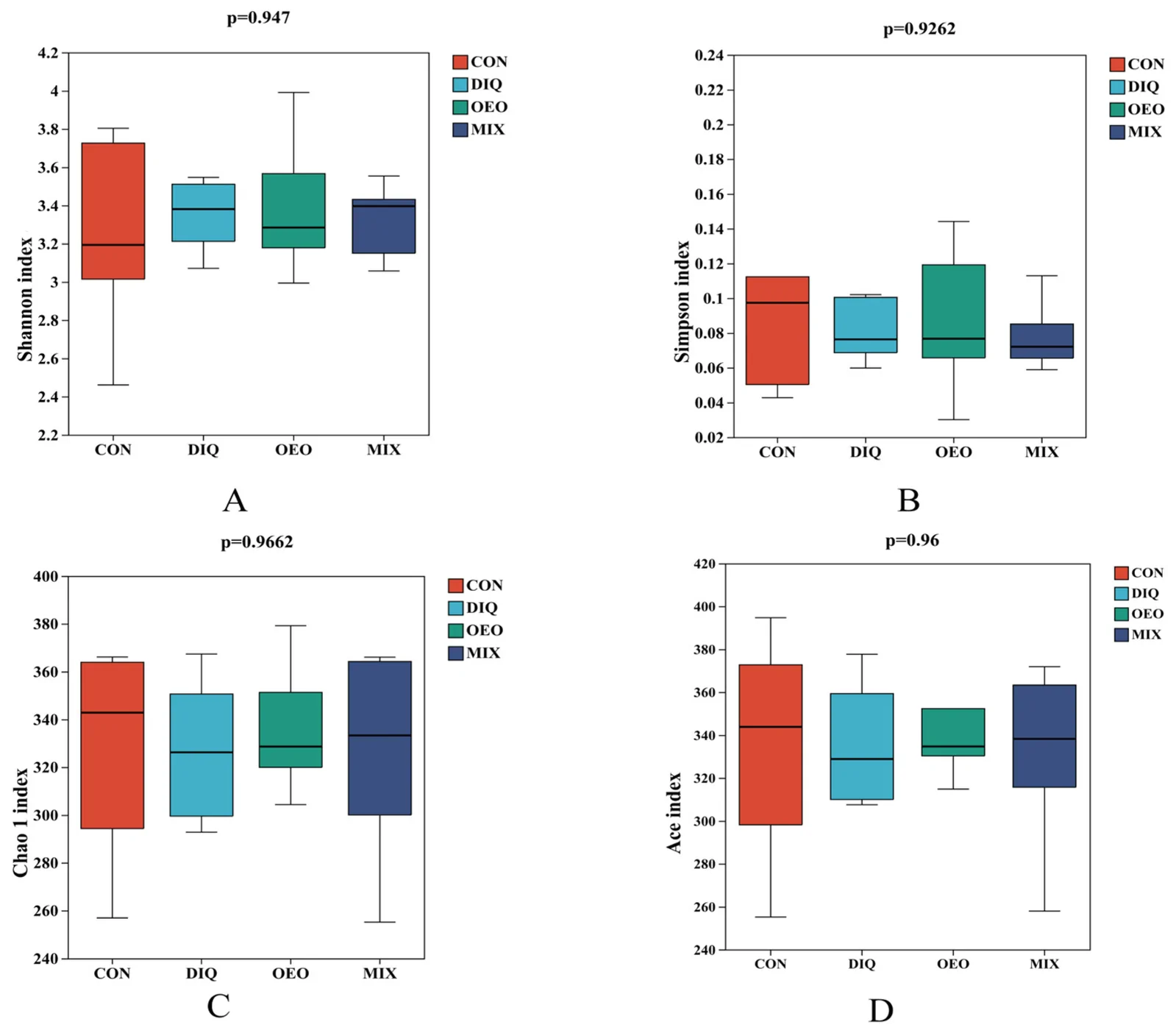
Effects of OEO and Diquat on the microbial alpha diversity of calf rumen. Note: (**A**) Shannon index; (**B**) Simpson index; (**C**) Chao1 index; and (**D**) ACE index. Data are shown for six calves per group (*n* = 6). Differences among the four treatment groups were assessed using global Kruskal–Wallis tests.

Alpha diversity analysis (including Chao1, Shannon, Simpson, and ACE indices) revealed no significant differences between treatment groups (*p* > 0.05) (Figure 4A–D). These findings indicate that Diquat injection and OEO supplementation did not significantly alter the richness or evenness of the calf rumen microbial community.

#### 3.4.2. Microbial Relative Abundance

**Figure 5:**
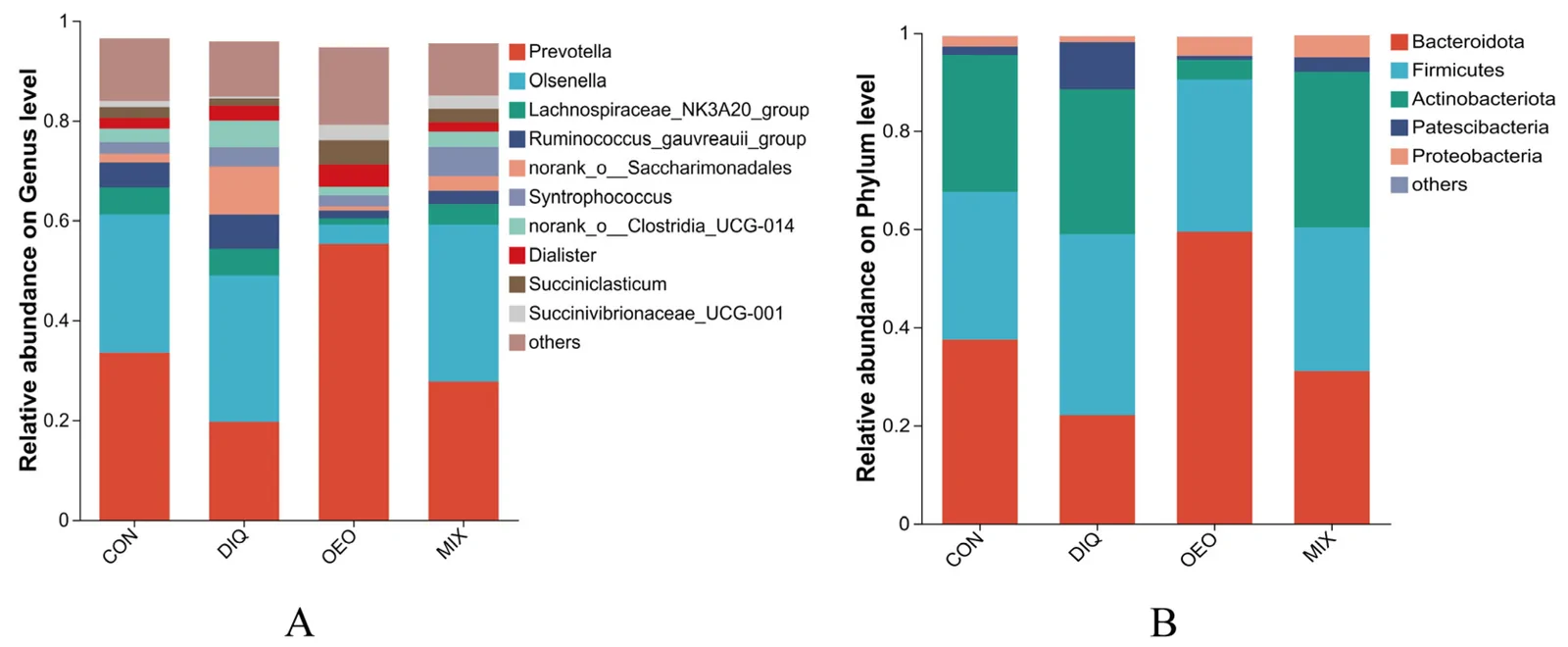
Effects of OEO and Diquat on the microbial abundance at the phylum and genus levels of calf rumen. Note: (**A**) Genus-level microbial abundance(top 10); (**B**) Phylum-level microbial abundance(top 5).

At the phylum level, the dominant phyla were Bacteroidetes, Firmicutes, Actinobacteria, Patescibacteria, and Proteobacteria (Table 7, Figure 5B). Notably, the relative abundance of Patescibacteria was significantly higher in the DIQ group compared to other groups (*p* < 0.05). At the genus level, the top ten genera by relative abundance comprised: *Prevotella*, *Olsenella*, Lachnospiraceae_NK3A20_group, Ruminococcus_gauvreauii_group, norank_o__Saccharimonadales, Syntrophococcus, *norank_o__Clostridia_UCG-014*, Dialister, Succiniclasticum, and *Succinivibrionaceae_UCG-001* (Table 7, Figure 5A). Notably, the abundance of *norank_o__Saccharimonadales* was also significantly higher in the DIQ group than in other groups (*p* < 0.05).

#### 3.4.3. LEfSe Analysis

**Figure 6:**
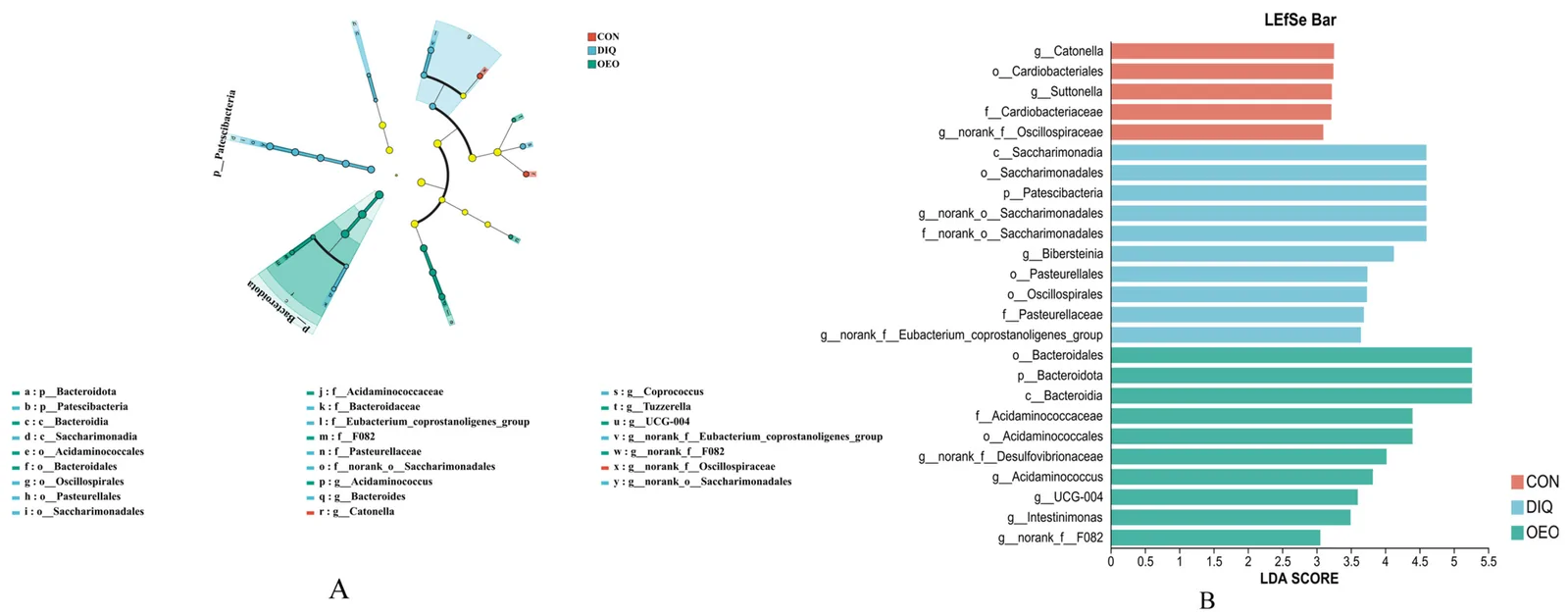
Effects of OEO and Diquat on Rumen Microbial Communities LEfSe Analysis. Note: (**A**) LEfSe cladogram showing the taxonomic distribution of differentially abundant taxa; (**B**) LDA score bar plot of differential taxa. Taxa with *p* < 0.05 and an LDA score > 3.0 were considered significantly different among groups.

Through LEfSe analysis (LDA ≥ 3, *p* < 0.05), biomarkers exhibiting significant intergroup differences were further screened (Figure 6B). Biomarkers in the CON group included *g__Catonella*, o__Cardiobacteriales, *g__Suttonella*, among others (5 Biomarkers). The DIQ group enriched 10 biomarkers, primarily comprising taxa associated with the order Saccharimonadales and phylum Patescibacteria, alongside o__Pasteurellales and others. The OEO group also identified 10 markers, predominantly belonging to the orders Bacteroidales and Acidaminococcales. No significantly different markers were detected in the MIX group. Multilevel species hierarchy trees revealed that the primary differential species originated from o_Oscillospirales, p_Patescibacteria, o__Pasteurellales, and f_Bacteroidaceae (Figure 6A).

#### 3.4.4. KEGG Pathway Functional Prediction

**Figure 7:**
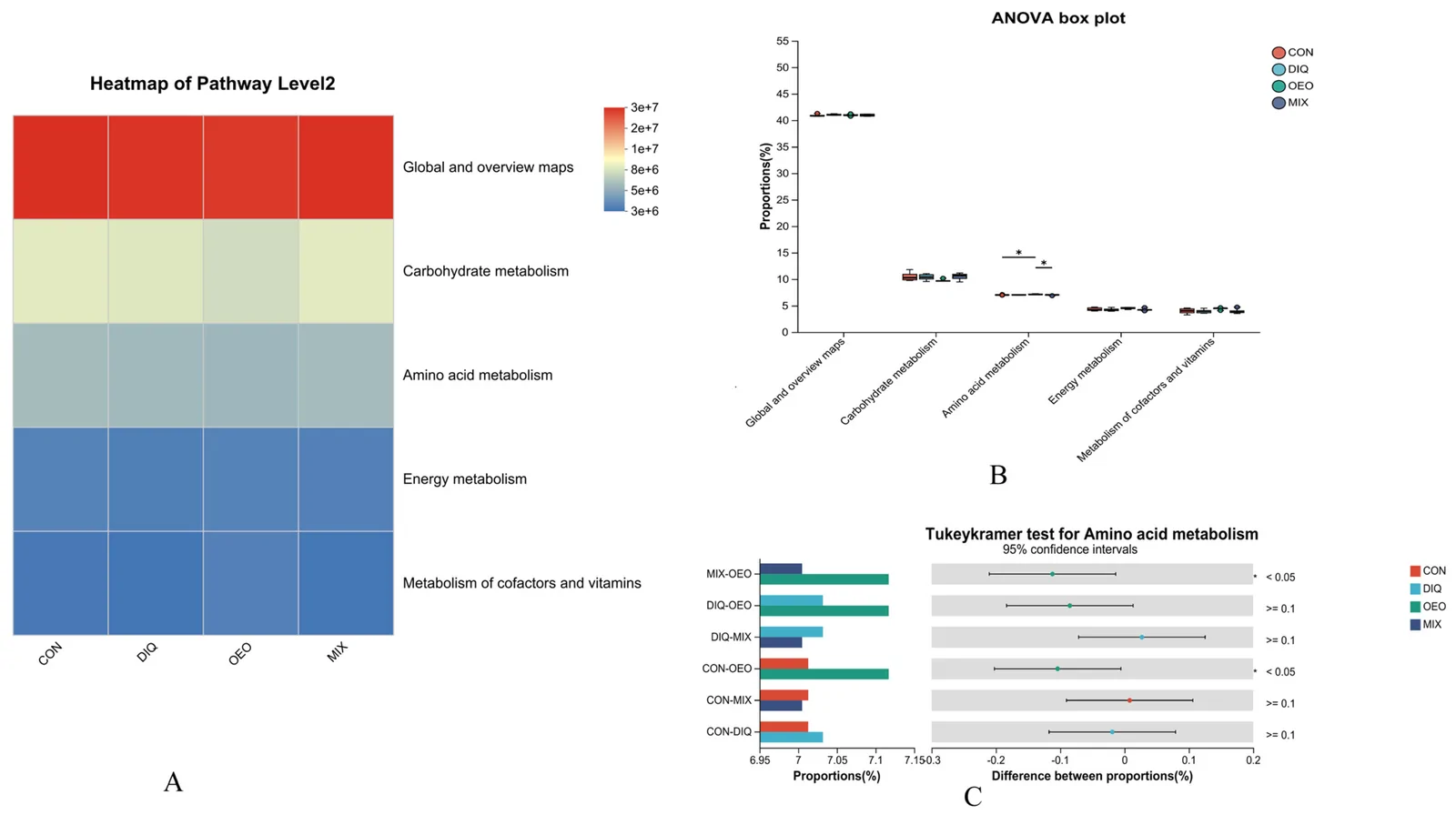
Functional Prediction of Rumen Microbiota Based on KEGG Pathways. Note: (**A**) PICRUSt2-predicted KEGG Level-2 functional profiles; (**B**) one-way ANOVA box plot of selected predicted functions; (**C**) Tukey–Kramer pairwise comparison for the predicted amino acid metabolism category. An asterisk (*) indicates a significant difference between groups (*p* < 0.05).

Functional potential of the rumen microbiota was predicted using PICRUSt2 (Figure 7A–C). KEGG Pathway analysis indicated that core functions of the four rumen microbial groups centred on metabolism, genetic information processing, and environmental information processing. Notably, amino acid metabolism-related pathways were significantly enriched in the OEO group, exhibiting markedly higher predicted abundance than both the CON (*p* < 0.05).

### 3.5. Rumen Fluid Metabolomics Analysis

**Figure 8:**
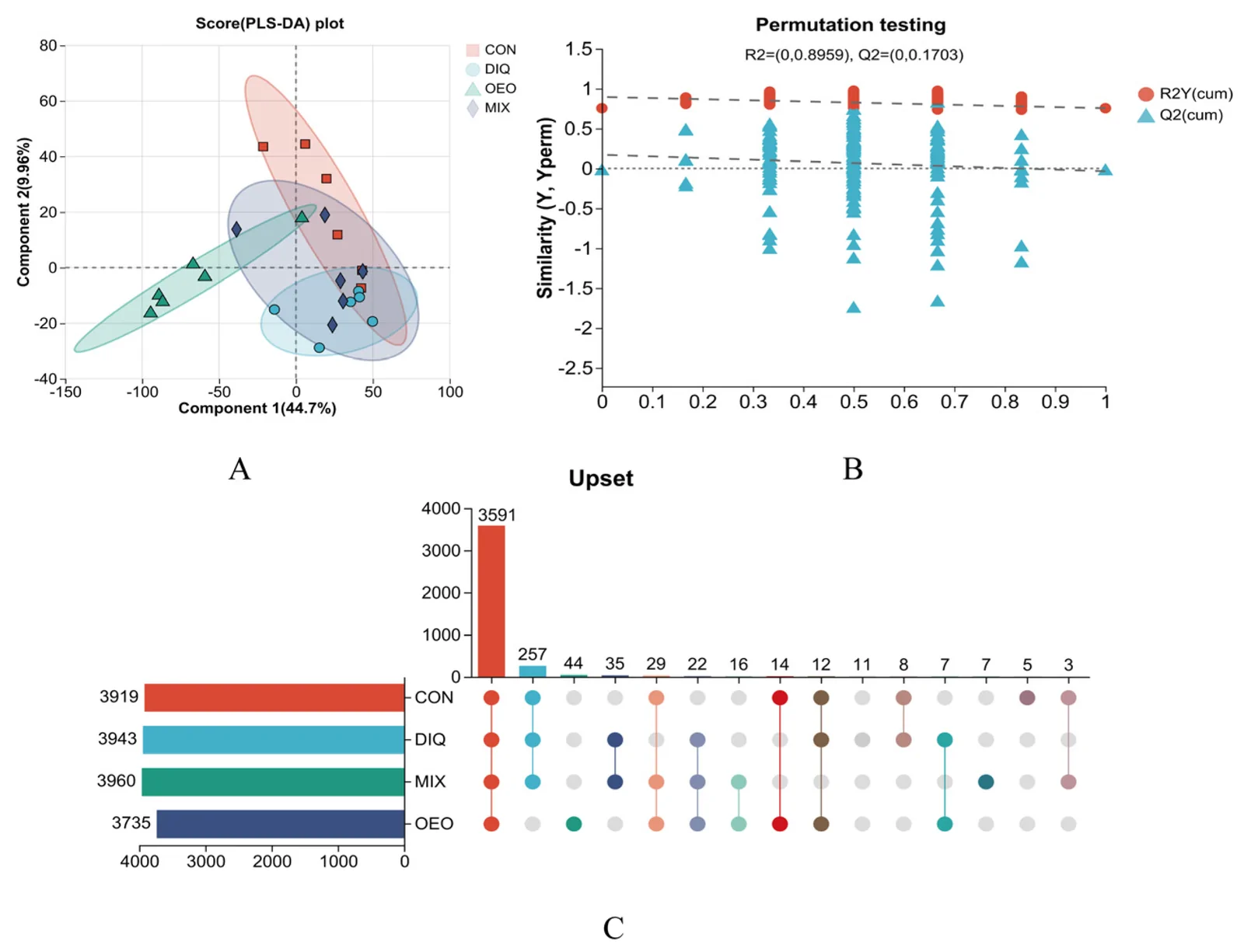
Multivariate analysis and set-intersection summary of rumen metabolite profiles. Note: (**A**) PLS-DA score plot; (**B**) PLS-DA permutation test; (**C**) UpSet plot showing intersections of detected metabolites among treatment groups. In panel B, the dashed lines represent the regression lines of R2Y(cum) and Q2(cum), respectively.

Non-targeted metabolomics was employed to analyse metabolites in calf rumen fluid. The Partial Least Squares Discriminant Analysis(PLS-DA) score plot demonstrated partial separation among the four sample groups (CON, DIQ, OEO, and MIX), indicating differences in metabolite composition between treatment groups (Figure 8A). Upset plot analysis revealed 3591 metabolites common to all four groups, with 5, 11, 44, and 7 differentially expressed metabolites specific to the CON, DIQ, OEO, and MIX groups respectively (Figure 8C).

#### 3.5.1. Differential Metabolite Profiles

**Figure 9:**
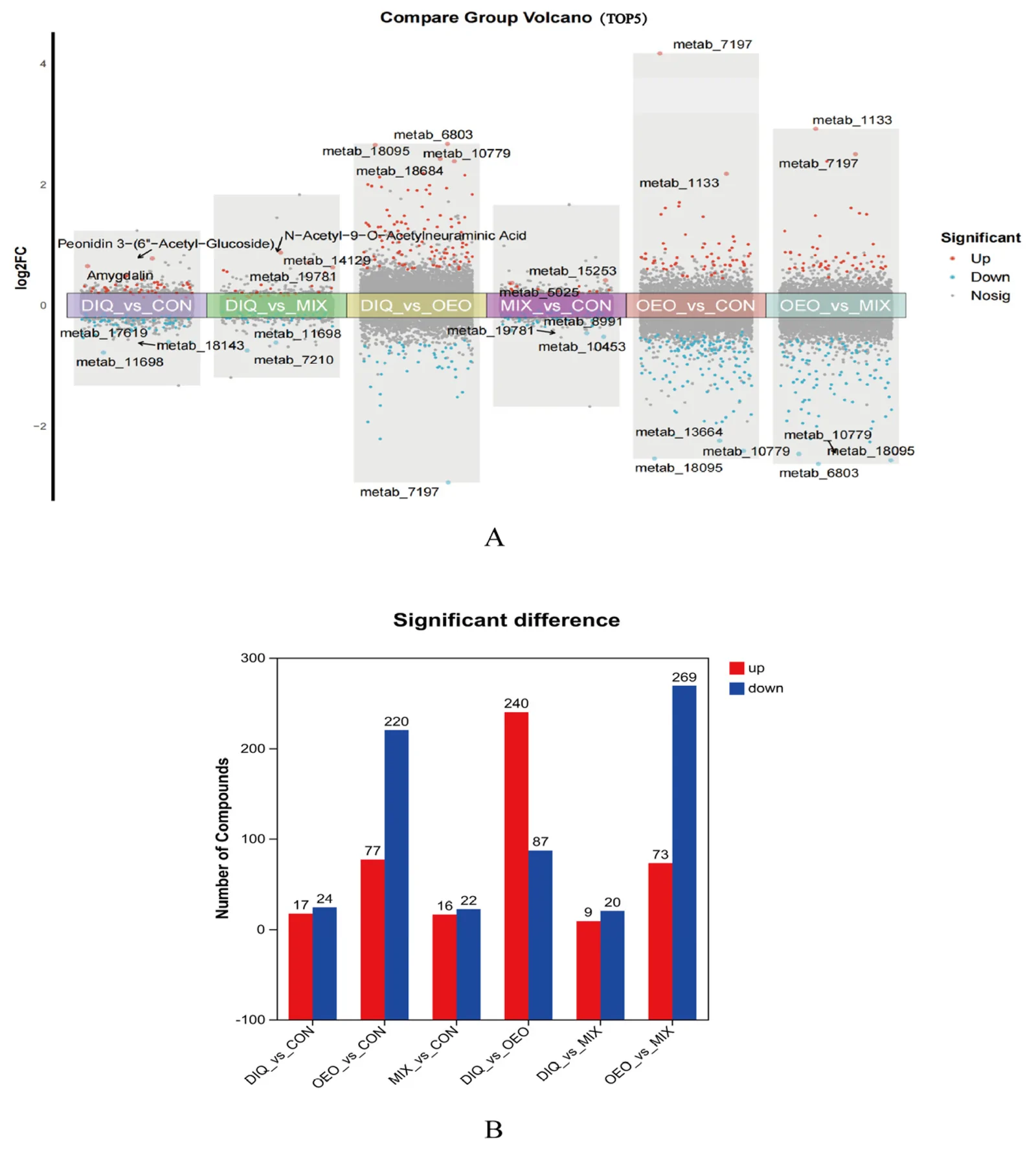
Number of Differentially Metabolized Compounds Among Groups Following OEO and Diquat Treatment. Note: (**A**) Volcano plots for pairwise comparisons; (**B**) numbers of upregulated and downregulated differential metabolites. Differential metabolites were defined by VIP > 1 and *p* < 0.05; direction was determined from the sign of log_2_FC (positive, upregulated; negative, downregulated) for the first group relative to the second group.

Candidate differential metabolites between groups were screened using the Orthogonal Partial Least Squares Discriminant Analysis (OPLS-DA) model (VIP > 1) combined with nominal two-tailed *t*-tests (*p* < 0.05). Compared with the CON group, the DIQ group exhibited 41 candidate differential metabolites, including 17 with higher abundance and 24 with lower abundance. When comparing the OEO group with the CON group, a total of 297 candidate differential metabolites were identified, including 77 with higher abundance and 220 with lower abundance. Compared with the OEO group, the DIQ group exhibited 240 candidate differential metabolites with higher abundance and 87 with lower abundance. When compared with the MIX group, the DIQ group showed 9 candidate differential metabolites with higher abundance and 20 with lower abundance. The OEO group, when compared with the MIX group, demonstrated 73 candidate differential metabolites with higher abundance and 269 with lower abundance (Figure 9A,B). Corresponding FDR-adjusted *p* values were calculated for these comparisons to provide a more conservative assessment of statistical evidence.

#### 3.5.2. KEGG Pathway Enrichment Analysis of Differential Metabolites

**Figure 10:**
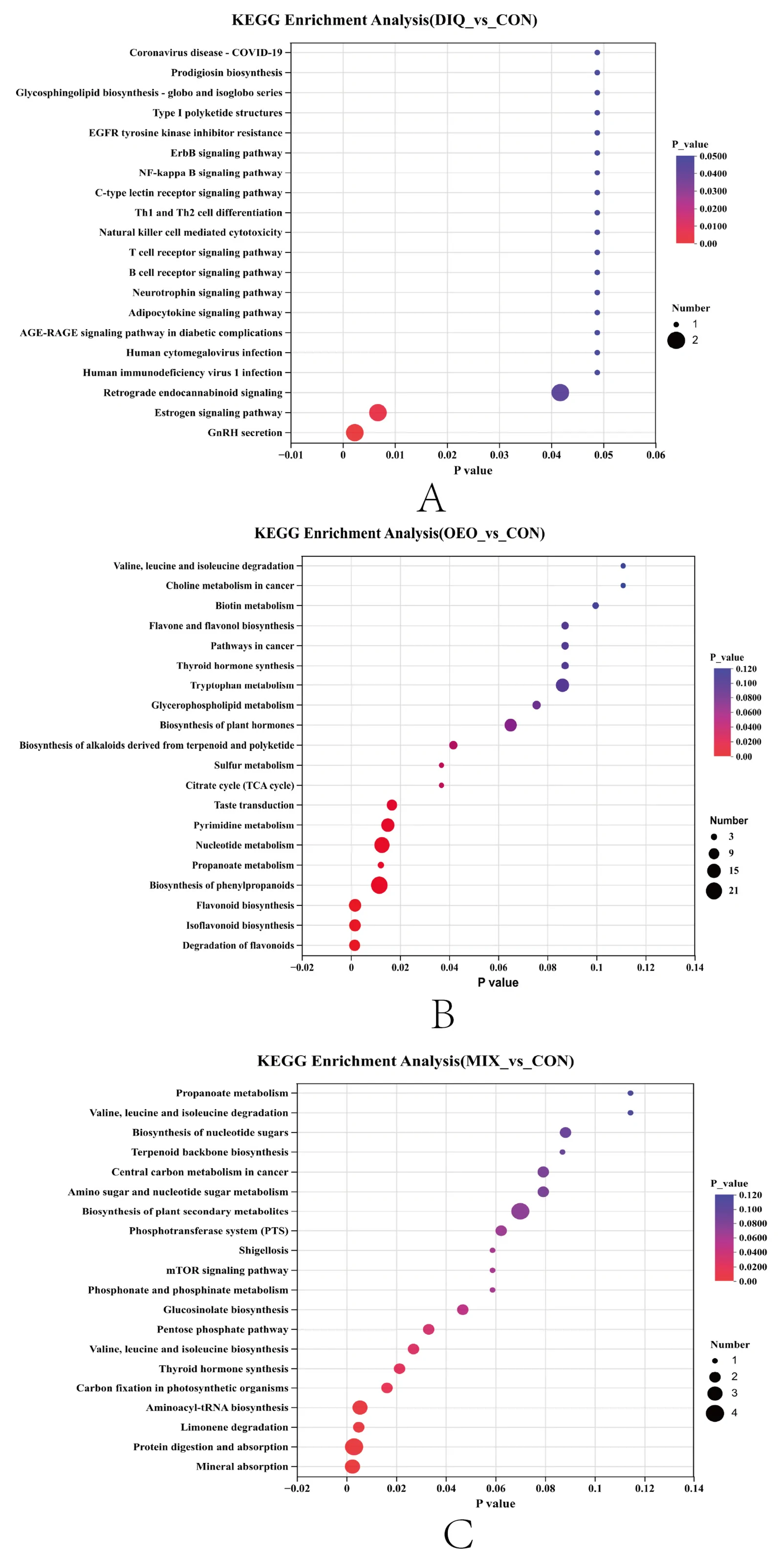
KEGG pathway-enrichment analysis of differential rumen metabolites. Note: (**A**) Top 20 KEGG enrichment results for DIQ versus CON; (**B**) top 20 results for OEO versus CON; (**C**) top 20 results for MIX versus CON.

Kyoto Encyclopedia of Genes and Genomes (KEGG) pathway-enrichment analysis identified treatment-associated differences in metabolite-to-pathway annotations (Figure 10A–C). In the DIQ versus CON comparison, enriched categories included NF-κB signalling, T cell receptor signalling, AGE-RAGE signalling, and ErbB signalling, together with several KEGG disease-labelled categories such as coronavirus disease (COVID-19) and cytomegalovirus infection (Figure 10A). In the OEO versus CON comparison, enriched annotations mainly involved plant-secondary-metabolite biosynthesis, including flavonoid and phenylpropanoid pathways, together with energy- and sulphur-metabolism-related pathways (Figure 10B). In the MIX versus CON comparison, enriched annotations included the pentose phosphate pathway, amino acid biosynthesis, and plant-secondary-metabolism-related pathways (Figure 10C).

#### 3.5.3. Correlation Analysis of Rumen Metabolites with Oxidative Stress and Inflammatory Factors

**Figure 11:**
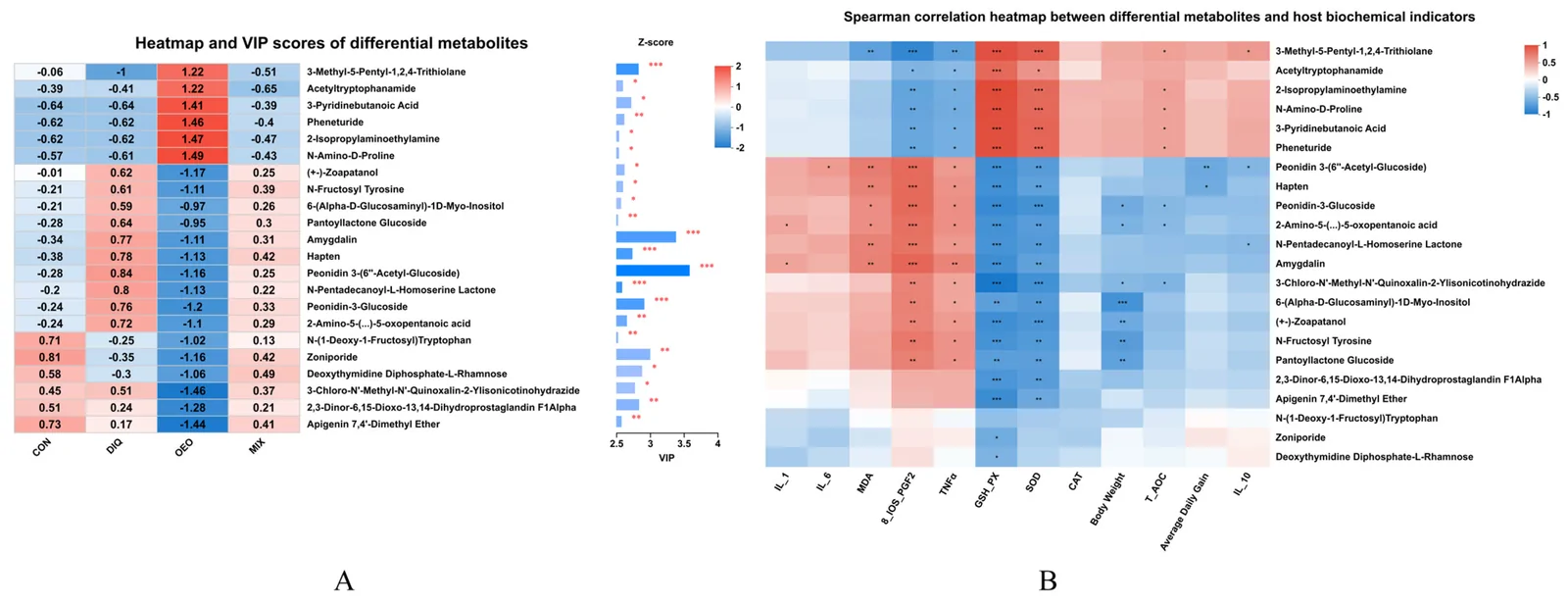
Expression patterns of selected differential rumen metabolites and their correlations with growth, serum antioxidant, oxidative-damage, and inflammatory indices. Note: (**A**) Heatmap of the Z-score-normalized abundance and VIP scores of selected differential metabolites (VIP > 2.5); (**B**) Spearman correlation heatmap between selected metabolites and host phenotypes. In panel A, the color scale represents Z-scores, with red indicating higher and blue indicating lower normalized abundance. In panel B, red and blue indicate positive and negative Spearman correlations, respectively. * *p* < 0.05, ** *p* < 0.01, *** *p* < 0.001.

To investigate the association between rumen metabolites and host oxidative stress and inflammatory status, correlation analysis was performed (Figure 11B). The results showed that 3-Methyl-5-Pentyl-1,2,4-Trithiolane, Acetyltryptophanamide, 2-Isopropylaminoethylamine, N-Amino-D-Proline, 3-Pyridinebutanoic Acid, and Pheneturide exhibited significant positive correlations with antioxidant markers (SOD, CAT, and T-AOC; ρ > 0.5), while showing significant negative correlations with the oxidative damage marker 8-iso-PGF2α and the pro-inflammatory cytokine TNF-α (ρ < −0.5). In contrast, Peonidin 3-(6″-Acetyl-Glucoside), Hapten, Peonidin-3-Glucoside,2-Amino-5-((1-((Carboxymethyl)Amino)-3-(Methylthio)-1-Oxopropan-2-Yl) Amino)-5-Oxopentanoic Acid, N-Pentadecanoyl-L-Homoserine Lactone, Amygdalin, 3-Chloro-N′-Methyl-N′-Quinoxalin-2-Ylisonicotinohydrazide,6-(Alpha-D-Glucosaminyl)-1D-Myo-Inositol, (+−)-Zoapatanol, N-Fructosyl Tyrosine, and Pantoyllactone Glucoside showed significant negative correlations with antioxidant markers (SOD, CAT, and T-AOC; ρ < −0.5), and significant positive correlations with the oxidative damage marker 8-iso-PGF2α, body weight, and the pro-inflammatory cytokine TNF-α (ρ > 0.5) (Figure 11B).

Notably, metabolites positively correlated with antioxidant indices and negatively correlated with oxidative-damage or inflammatory indices showed distinct abundance patterns among the treatment groups, whereas metabolites showing the opposite correlation pattern exhibited different abundance profiles (Figure 11A).

## 4. Discussion

### 4.1. Growth Performance

This study evaluated the protective effects associated with OEO pre-supplementation before a diquat-induced oxidative challenge in calves. Because OEO supplementation preceded diquat administration by 14 days, the present findings primarily reflect a preconditioning or prophylactic effect rather than therapeutic reversal of established oxidative injury. As a potent inducer [9], diquat triggers oxidative stress, leading to lipid peroxidation, macromolecule damage, liver injury, and impaired growth [4,5,6]. Our prior research demonstrated that intraperitoneal injection of 8 mg/kg BW diquat significantly elevated calf blood levels of ALT, AST, MDA, COR, and NOR, while markedly reducing concentrations of SOD, GSH-PX, T-AOC, and CAT [25]. This study similarly found that intraperitoneal injection of diquat decreased calf blood SOD, T-AOC, and GSH-Px activities, while significantly increasing ALT, AST, and MDA concentrations, confirming the successful induction of oxidative stress in calves. Previous studies have demonstrated that oxidative stress impairs growth performance in calves [25], weaned piglets [36,37], and poultry [38], consistent with the present findings. Diquat injection markedly reduced calf ADG and exerted a phase-dependent effect on DMI, with a significant reduction observed during D15–21 but not over the overall D1–21 period. These changes may be related to ROS generated during diquat metabolism, which can damage intestinal epithelial cell membranes, impair nutrient absorption, and compromise intestinal function [39,40,41,42,43], thereby adversely affecting growth performance. Notably, OEO supplementation independently improved ADG and DMI during D15–21, consistent with our previous studies [22,23,44,45]. Phenolic compounds in OEO (carvacrol, thymol) help maintain epithelial integrity, enhance digestive function [46], promote villus development [47], and its aroma may stimulate appetite [48], which may contribute to the overall improvement in feed intake associated with OEO supplementation.

### 4.2. Blood Parameters

An imbalanced redox state leads to ROS accumulation, oxidative stress, and damage to biomolecules, producing byproducts like MDA and 8-Iso-PGF2α [49,50]. The body relies on enzymatic antioxidants (SOD, GSH-Px, CAT) and T-AOC to maintain equilibrium [51,52]. MDA is a biomarker of lipid peroxidation [53], and 8-Iso-PGF2α results from free radical attack on membrane phospholipids [50]. Liver enzymes (AST, ALT, ALP) indicate hepatic injury [54]. Consistent with previous studies [25,55,56], diquat challenge significantly decreased serum SOD, GSH-Px, and T-AOC and increased 8-iso-PGF2α, AST, and ALT on D15 and D21. Serum MDA was significantly increased by diquat on D15, whereas no significant diquat effect on MDA was detected on D21. OEO supplementation was associated with improved antioxidant status and lower oxidative-damage and liver-injury indices, although the magnitude of these responses varied between D15 and D21. OEO’s active components, carvacrol and thymol, possess potent antioxidant properties [48,57,58]. Their phenolic structures not only directly scavenge free radicals and provide hydrogen atoms to neutralise hydrogen peroxide and superoxide anions [59], but also inhibit metal-catalysed radical generation by chelating metal ions [60]. This mitigates oxidative damage to biomolecules and cell membranes, thereby reducing serum MDA and 8-Iso-PGF2α levels. On D15, diquat challenge increased serum IL-1β, IL-6, and TNF-α and reduced IL-10, whereas OEO supplementation partially reversed these changes. On D21, significant treatment effects remained for IL-1β and IL-10, while IL-6 and TNF-α were no longer significantly affected. Oxidative stress frequently correlates with pro-inflammatory cytokine expression [61]. Previous studies have shown that diquat challenge is associated with increased expression or concentrations of pro-inflammatory cytokines, including IL-1β, IL-6, and TNF-α, in weaned piglets [62]. Zhou et al. found that Diquat stress induced elevated serum TNF-α and IL-6 levels in broiler chickens [63]. This aligns with our findings. When subjected to ROS assault, immune cells respond to tissue damage induced by diquat by infiltrating the injured site during differentiation and producing high levels of pro-inflammatory cytokines such as IL-1β, IL-6, and TNFα [64]. This enables immune cells to better access the injury site for reparative action. OEO enhances anti-inflammatory pathways by inhibiting the activation of Mitogen-activated protein kinase(MAPK) and NF-κB pathways [21], thereby reducing serum levels of pro-inflammatory factors.

### 4.3. Nrf2-Related Antioxidant Gene Expression

The Nrf2 regulatory network is a central component of cellular antioxidant defence [65,66,67]. *Keap1* participates in the regulation of Nrf2 stability, whereas genes such as *GCLC*, *GCLM*, *HMOX1*, and *SOD1* are involved in antioxidant and redox responses [68,69,70]. In the present study, diquat was associated with higher *Keap1* mRNA expression and lower *GCLC* and *GCLM* expression, whereas OEO supplementation was associated with lower *Keap1* expression and higher *HMOX1*, *GCLC*, *GCLM*, and *SOD1* expression. These findings suggest that OEO treatment may have altered the expression of related Nrf2 antioxidant genes. Zou et al. reported that OEO induced Nrf2-related antioxidant responses in IPEC-J2 cells, including elevated *SOD1* and glutathione-related responses [19], providing supporting evidence for the involvement of Nrf2-related antioxidant responses. OEO supplementation was associated with increased expression of *HMOX1*, *GCLC*, *GCLM*, and *SOD1*, which may contribute to improved antioxidant capacity. However, the present study only evaluated the mRNA expression of Nrf2-related antioxidant genes and did not assess Nrf2 protein abundance, nuclear translocation, phosphorylation status, or ARE-dependent transcriptional activity. Therefore, these findings indicate altered expression of Nrf2-related antioxidant genes but do not provide direct evidence of Nrf2 signaling pathway activation. Further studies incorporating protein-level and functional analyses are required to confirm the involvement of Nrf2 signaling in the antioxidant effects of OEO.

### 4.4. Rumen Microbiome

A stable rumen microbiome is closely associated with calf development and health. Research indicates that oxidative stress can disrupt intestinal microbial homeostasis, inducing dysbiosis and promoting the enrichment of pro-inflammatory or pathogenic microbial populations, thereby exacerbating systemic inflammatory responses and metabolic disorders [71]. Therefore, it is necessary to investigate whether diquat mediates oxidative stress by altering rumen microbial community structure, and whether oregano essential oil supplementation exerts a protective effect by improving microbial homeostasis. This study employed 16S rRNA sequencing to investigate the effects of Diquat injection and OEO supplementation on calf rumen microbiota. Findings revealed that neither Diquat injection nor OEO supplementation significantly altered α-diversity in calf rumen microbiota, Although neither diquat challenge nor OEO supplementation significantly altered α-diversity, an overall treatment-associated difference in β-diversity was detected, with a relatively modest effect size. These findings indicate treatment-associated differences in rumen microbial community composition following OEO supplementation and diquat challenge. However, the present design does not distinguish direct effects of OEO on the rumen microbiota from indirect effects associated with changes in feed intake [72]. In the DIQ group, the phylum *Patescibacteria* and the genus *norank_o__Saccharimonadales* under the order Saccharimonadales showed significant enrichment. Previous human studies have reported associations of Patescibacteria with gastric cancer and Saccharimonadales with oral inflammatory conditions [73,74]. However, these associations do not establish pathogenic or pro-inflammatory roles for these taxa in the calf rumen. Their enrichment therefore indicates a treatment-associated shift in rumen microbial composition under diquat challenge, but its biological significance in calves remains to be determined. In contrast, the OEO group exhibited enrichment of several microbial taxa. Bacteroidales have been associated with complex carbohydrate degradation, SCFA production, and immune-related functions [75]. Acidaminococcaceae and Acidaminococcus have been linked to amino acid metabolism [76], while Intestinimonas is known to produce butyrate [77]. In addition, g_norank_f_F082 has been reported to correlate positively with gas production, ruminal fermentation, and antioxidant capacity [78]. These findings indicate that OEO supplementation was associated with microbial shifts involving taxa previously linked to rumen metabolic functions; however, these functional roles were not directly evaluated in the present study. LEfSe analysis (LDA ≥ 3, *p* < 0.05) further identified several taxa enriched in the DIQ group, including *Saccharimonadales* (inflammatory mucosal diseases), *Pasteurellaceae* (cancer-related), and *Eubacterium_coprostanoligenes_group* (rheumatoid arthritis-related) [79]. Although these taxa have been associated with disease-related conditions in studies from other hosts or anatomical sites, their functional significance in the calf rumen remains uncertain and requires direct validation. the OEO group showed differential enrichment of taxa mainly belonging to Bacteroidales and Acidaminococcales, indicating treatment-associated shifts in rumen microbial composition. Notably, no differential biomarkers were identified in the MIX group. Overall, OEO supplementation was associated with changes in rumen microbial composition under diquat challenge. Because OEO also affected feed intake, an intake-mediated contribution to these microbial differences cannot be excluded, and the biological significance of these changes requires further validation.

### 4.5. Rumen Metabolomics and Correlation Analysis

To investigate whether diquat challenge was associated with changes in ruminal metabolic profiles and whether OEO supplementation was accompanied by corresponding metabolic alterations, untargeted LC-MS metabolomics was performed. KEGG enrichment analysis showed that differential metabolites associated with diquat challenge were mapped to annotations related to NF-κB signaling, AGE-RAGE signaling, T cell receptor signaling, and other immune-related categories. These enrichment patterns indicate overlap between the differential metabolite profile and KEGG pathway annotations but do not demonstrate activation of the corresponding signaling pathways. In addition, several disease- and plant-related KEGG categories were identified. Because individual metabolites can be assigned to multiple KEGG pathways, these categories are likely to reflect shared metabolite annotations and should not be interpreted as direct evidence of disease-related or plant-specific pathway activity in the calf rumen. These findings are consistent with previous reports indicating that diquat challenge induces oxidative stress and inflammatory responses [10]. N-Fructosyl Tyrosine was among the putatively annotated metabolites associated with the DIQ group. Previous studies have linked AGE-related compounds with oxidative and inflammatory processes [80].

2,3-Dinor-6,15-Dioxo-13,14-Dihydroprostaglandin F1α, an oxidative metabolite of PGF_2_α, has been associated with oxidative processes in previous studies [81]. These metabolite changes were observed in association with the diquat challenge, but their biological significance requires further validation. Notably, although peonidin glycoside metabolites (e.g., peonidin 3-glucoside) have been reported to exhibit antioxidant and anti-inflammatory activities [82], their changes may reflect treatment-associated metabolic responses, although their specific roles in the calf rumen remain uncertain. Moreover, N-Pentadecanoyl-L-Homoserine Lactone has been linked to TRPV1/TRPA1-related responses in previous studies [83], but its functional significance in the calf rumen remains unclear. In contrast, the OEO and MIX groups also showed distinct metabolite profiles relative to the DIQ group. Differential metabolites in the OEO group were mapped to flavonoid-, phenylpropanoid-, and other metabolism-related KEGG annotations. Previous studies have suggested that these compounds may influence oxidative and inflammatory responses through ROS scavenging and regulation of antioxidant and inflammatory pathways [19,21]. The MIX group showed changes in differential metabolites together with enrichment of metabolite annotations related to the pentose phosphate pathway and amino acid biosynthesis. Notably, Apigenin 7,4′-Dimethyl Ether was increased in the MIX group and has been associated with NF-κB-related responses in previous studies [84]. However, further functional validation is required to determine whether this metabolite contributes directly to the effects associated with OEO supplementation. Amygdalin and Pantoyllactone Glucoside also differed among treatment groups; however, their functional relevance in the calf rumen was not directly assessed.

Correlation analysis identified associations between selected rumen metabolites and host antioxidant, oxidative-damage, and inflammatory indices. Metabolites upregulated in the DIQ group showed positive correlations with oxidative damage markers (MDA, 8-ISO-PGF2α) and pro-inflammatory cytokines (TNF-α), while exhibiting negative correlations with antioxidant enzyme activities (SOD, GSH-Px, T-AOC). These findings suggest that OEO supplementation is associated with changes in ruminal metabolic profiles, including metabolites related to phenolic compounds and flavonoid metabolism. These findings indicate associations between OEO-related ruminal metabolic changes and systemic antioxidant and inflammatory indices. However, because samples from all treatment groups were pooled for the correlation analysis, some of the observed associations may partly reflect between-group differences. Therefore, these correlations should be interpreted as exploratory associations rather than evidence of direct causal or mechanistic relationships. In addition, non-specific or low-confidence metabolite annotations were excluded from mechanistic interpretation because their biological significance remains uncertain [85]. Overall, targeted metabolite validation and additional analyses are needed to clarify the biological relevance of these metabolic changes and determine whether the observed associations are independent of treatment-group effects.

### 4.6. Study Limitations

Several limitations should be considered in the present study. First, hepatic RT-qPCR analysis was performed using three biological replicates per group, and technical replicates cannot replace biological replication. Second, Nrf2 protein abundance, nuclear translocation, phosphorylation status, and ARE activity were not evaluated; therefore, direct activation of the Nrf2 pathway requires further validation. Third, rumen microbiota were assessed only at the endpoint, and OEO also affected feed intake; thus, direct microbial effects cannot be clearly separated from intake-related effects. Rumen pH, volatile fatty acids, and ammonia nitrogen were not measured, so the functional consequences of the observed microbial shifts remain uncertain. In addition, 16S-based functional predictions and untargeted metabolite annotations require further validation. Finally, the relationships among rumen microbiota, metabolites, and host antioxidant responses remain associative.

## 5. Conclusions

This study systematically evaluated the protective effects of oregano essential oil (OEO) using a Diquat induced oxidative stress model in calves. Diquat successfully induced oxidative stress and inflammation, while altering the expression of hepatic Nrf2-related genes. Dietary supplementation with OEO improved feed intake, weight gain, and antioxidant enzyme activities, while alleviating oxidative damage and inflammatory responses. These effects were accompanied by changes in the expression levels of Nrf2-related antioxidant genes, including *HMOX1, GCLC, GCLM*, and *SOD1*, as well as alterations in rumen microbial composition and metabolite profiles. In conclusion, OEO pre-supplementation was associated with improved antioxidant status and altered microbial and metabolic responses after diquat challenge, supporting a potential prophylactic rather than therapeutic effect.

## Figures and Tables

**Table 1:** Ingredient composition of the pelleted concentrate and nutrient composition of the TMR (90:10 pelleted concentrate:oat hay).

**A. Ingredient composition of the pelleted concentrate (% of concentrate DM)**					
**Ingredient**	**%**	**Ingredient**	**%**	**Ingredient**	**%**
Corn	44.48	Soybean meal	24.50	Wheat bran	8.00
Cottonseed meal	4.80	Puffed soybeans	4.00	Whey powder	4.00
Molasses	4.00	Soy protein concentrate	2.00	CaCO_3_	1.40
VTM premix ^1^	1.00	Salt	0.80	Soybean oil	0.50
CaHPO_4_	0.30	MgO	0.20	Flavor	0.02
Total	100				
**B. Nutrient composition**					
**Nutrient**	**Pelleted concentrate**		**Oat hay**	**Basal TMR ^2^**	
Dry matter, % as-fed	88.0		89.6	88.1	
Crude protein, % DM	23.1		6.89	21.5	
Ether extract, % DM	3.85		1.85	3.65	
Ash, % DM	7.80		5.24	7.54	
Neutral detergent fiber, % DM	12.3		51.1	16.2	
Acid detergent fiber, % DM	5.79		26.3	7.84	
Calcium, % DM	0.90		0.24	0.83	
Phosphorus, % DM	0.60		0.11	0.55	

Note: ^1^ Vitamin trace mineral premix contains: Cu: 1000 mg/kg, Fe: 5600 mg/kg, Mn: 4200 mg/kg, Zn: 9000 mg/kg, I: 100 mg/kg, Se: 40 mg/kg, Co: 35 mg/kg, Vitamin A: 900,000 IU/kg, Vitamin D3: 400,000 IU/kg, Vitamin E: 6500 IU/kg. ^2^ The basal TMR consisted of 90% pelleted concentrate and 10% oat hay on a DM basis; its nutrient composition was calculated from the component values.

**Table 2:** The sequences of primers used for RT–qPCR.

Gene	Primer Sequences (5′→3′)	Product Length, bp
*Keap1*	F: TGTCCTCAACCGTCTGCTCTAC R: TCCGCCACTCGTTTCTCTCTG	98
*HMOX1*	F: ACCGCCTTCCTGCTCAACATC R: TCGCCTGTGAAGCTCTGGTG	105
*NQO1*	F: ATGAAGGAGGCTGCCATAGAGG R: CTGGAGATGACGGGATTGAAGTTC	95
*GPX4*	F: CTGGTCGTGCTCGGCTTCC R: CGCCTGGTCGGACGTACTTC	97
*GCLC*	F: GCCTCCTCCTCCAAACTCTGAC R: GCACCACGAACACCACATACG	104
*GCLM*	F: TGATGCCACCTGATTTGACTGC R: GTGCTTCTTGGAAACTTGCTTCAG	105
*SOD1*	F: GCTGTACCAGTGCAGGTCCTC R: TGTCACATTGCCCAGGTCTCC	98
*GAPDH*	F: AAAGTGGACATCGTCGCCAT R: CCGTTCTCTGCCTTGACTGT	116

Note: F Forward, R Reverse, Keap1 Kelch-like ECH-associated protein 1, HMOX1 Heme oxygenase-1, NQO1 NAD(P)H quinone oxidoreductase1,GSH-PX(GPX4) Glutathione Peroxidase 4, GCLC Glutamate-Cysteine Ligase, Catalytic Subunit, GCLM Glutamate-Cysteine Ligase Modifier Subunit, SOD1 Superoxide Dismutase 1, GAPDH Glyceraldehyde-3-Phosphate Dehydrogenase.

**Table 3:** Effects of oregano essential oil supplementation and diquat challenge on growth performance in calves.

	Group				*p*-Value ^1^		
Parameter	CON	DIQ	OEO	MIX	OEO	Diquat	OEO × Diquat
Calves, n	6	6	6	6	—	—	—
BW, kg							
D 1	117.67 ± 7.07	115.00 ± 4.63	118.08 ± 5.95	117.83 ± 6.91	—	—	—
D 15	126.08 ± 6.94	124.17 ± 4.59	128.42 ± 6.07	128.50 ± 6.97	0.021	0.440	0.771
D 21	134.67 ± 6.59	128.58 ± 4.56	139.08 ± 6.10	135.00 ± 7.08	<0.001	0.001	0.859
ADG, kg/d							
D 1–14	0.60 ± 0.04	0.65 ± 0.04	0.74 ± 0.06	0.76 ± 0.05	0.013	—	—
D 15–21	1.23 ± 0.07 ^ab^	0.63 ± 0.06 ^c^	1.52 ± 0.11 ^a^	0.93 ± 0.05 ^bc^	0.002	<0.001	1.000
D 1–21	0.81 ± 0.04 ^bc^	0.65 ± 0.05 ^c^	1.00 ± 0.05 ^a^	0.82 ± 0.02 ^b^	<0.001	<0.001	0.815
DMI, kg/d							
D 1–14	3.86 ± 0.20	3.96 ± 0.13	4.43 ± 0.14	4.21 ± 0.30	0.024	—	—
D 15–21	4.60 ± 0.15 ^ab^	4.01 ± 0.11 ^b^	4.82 ± 0.19 ^a^	4.54 ± 0.22 ^ab^	0.023	0.010	0.324
D 1–21	4.12 ± 0.18	3.98 ± 0.11	4.57 ± 0.15	4.32 ± 0.27	0.024	0.243	0.747

Note: CON, basal diet + saline; DIQ, basal diet + diquat challenge; OEO, basal diet + oregano essential oil; MIX, oregano essential oil + diquat challenge. ^1^ *p*-values correspond to the main effects of oregano essential oil supplementation (OEO), diquat challenge, and the OEO × Diquat interaction in the 2 × 2 factorial model. Within a row, means with different superscript letters differ significantly (*p* < 0.05). The same below.

**Table 4:** Effects of oregano essential oil supplementation and diquat challenge on serum liver-function indices in calves.

	Group				*p*-Value ^1^		
Parameter	CON	DIQ	OEO	MIX	OEO	Diquat	OEO × Diquat
Calves, n	6	6	6	6	—	—	—
AST, U/L							
D 1	95.39 ± 4.86	107.34 ± 2.79	104.25 ± 7.42	109.52 ± 4.96	—	—	—
D 15	95.59 ± 2.32 ^bc^	119.53 ± 4.93 ^a^	87.34 ± 3.14 ^c^	105.42 ± 3.31 ^b^	0.002	<0.001	0.578
D 21	91.09 ± 2.37 ^bc^	105.27 ± 2.27 ^a^	84.46 ± 1.67 ^c^	95.23 ± 2.81 ^b^	0.003	<0.001	0.569
ALT, U/L							
D 1	21.53 ± 0.85	20.85 ± 0.82	22.06 ± 0.82	20.69 ± 0.53	—	—	—
D 15	22.19 ± 1.50 ^b^	30.71 ± 0.96 ^a^	19.77 ± 0.69 ^b^	26.67 ± 1.45 ^a^	0.007	<0.001	0.404
D 21	26.97 ± 0.62 ^ab^	31.21 ± 2.58 ^a^	22.49 ± 0.23 ^b^	25.97 ± 2.17 ^ab^	0.014	0.081	0.752
ALP, U/L							
D 1	2.11 ± 0.40	1.61 ± 0.17	2.55 ± 0.16	2.23 ± 0.16	—	—	—
D 15	3.02 ± 0.34	4.05 ± 0.33	3.15 ± 0.31	3.67 ± 0.27	0.619	0.035	0.411
D 21	3.46 ± 0.39	3.91 ± 0.30	3.03 ± 0.33	3.35 ± 0.20	0.194	0.276	0.833

**Table 5:** Effects of oregano essential oil supplementation and diquat challenge on serum antioxidant and oxidative-stress indices in calves.

	Group				*p*-Value ^1^		
Parameter	CON	DIQ	OEO	MIX	OEO	Diquat	OEO × Diquat
Calves, n	6	6	6	6	—	—	—
T-AOC, U/mL							
D 1	10.23 ± 0.20	10.61 ± 0.30	9.92 ± 0.56	10.91 ± 0.38	—	—	—
D 15	10.98 ± 0.37 ^a^	8.81 ± 0.24 ^b^	11.90 ± 0.25 ^a^	11.28 ± 0.39 ^a^	<0.001	<0.001	0.058
D 21	11.88 ± 0.23 ^a^	10.31 ± 0.22 ^b^	11.36 ± 0.23 ^ab^	10.51 ± 0.20 ^b^	0.525	<0.001	0.142
CAT, U/mL							
D 1	47.47 ± 1.00	45.30 ± 0.27	48.28 ± 0.29	47.60 ± 0.67	—	—	—
D 15	51.22 ± 1.09 ^b^	57.25 ± 0.45 ^ab^	62.69 ± 0.34 ^a^	62.30 ± 3.20 ^a^	0.002	0.142	0.088
D 21	53.25 ± 1.64	48.05 ± 1.30	53.49 ± 3.13	49.99 ± 0.61	0.191	0.015	0.379
GSH-Px, U/mL							
D 1	164.67 ± 1.60	163.82 ± 2.10	160.59 ± 3.22	165.51 ± 2.01	—	—	—
D 15	207.90 ± 2.27 ^b^	161.32 ± 3.39 ^c^	290.75 ± 9.39 ^a^	188.56 ± 1.40 ^b^	0.006	<0.001	0.052
D 21	167.20 ± 3.17 ^a^	127.40 ± 1.63 ^c^	170.24 ± 3.37 ^a^	142.60 ± 2.53 ^b^	<0.001	<0.001	<0.001
SOD, U/mL							
D 1	69.69 ± 1.50	71.54 ± 1.34	68.38 ± 1.64	73.40 ± 2.29	—	—	—
D 15	71.81 ± 1.28 ^b^	63.75 ± 0.82 ^c^	86.68 ± 1.00 ^a^	80.30 ± 1.52 ^a^	<0.001	<0.001	0.300
D 21	73.61 ± 0.59 ^b^	66.76 ± 0.57 ^c^	90.23 ± 0.91 ^a^	71.85 ± 1.25 ^b^	<0.001	<0.001	<0.001
MDA, nmol/mL							
D 1	3.69 ± 0.21	3.57 ± 0.12	3.42 ± 0.17	3.35 ± 0.16	—	—	—
D 15	3.69 ± 0.11 ^b^	6.03 ± 0.15 ^a^	3.80 ± 0.14 ^b^	4.35 ± 0.11 ^b^	0.031	0.128	0.816
D 21	3.61 ± 0.12 ^ab^	3.37 ± 0.12 ^b^	3.84 ± 0.13 ^a^	3.67 ± 0.15 ^ab^	<0.001	<0.001	<0.001
8-iso-PGF2α, pg/mL							
D 1	115.71 ± 2.90	114.04 ± 3.65	122.01 ± 3.61	112.00 ± 2.74	—	—	—
D 15	116.21 ± 2.00 ^c^	170.57 ± 2.26 ^a^	106.37 ± 4.28 ^c^	141.34 ± 2.15 ^b^	<0.001	<0.001	0.004
D 21	116.32 ± 1.64 ^bc^	153.60 ± 1.49 ^a^	111.81 ± 5.05 ^c^	131.90 ± 5.65 ^b^	0.004	<0.001	0.136

**Table 6:** Effects of oregano essential oil supplementation and diquat challenge on serum inflammatory cytokines in calves.

	Group				*p*-Value ^1^		
Parameter	CON	DIQ	OEO	MIX	OEO	Diquat	OEO × Diquat
Calves, n	6	6	6	6	—	—	—
IL-1β, pg/mL							
D 1	21.55 ± 0.56	21.16 ± 0.38	23.08 ± 1.40	21.38 ± 0.82	—	—	—
D 15	21.34 ± 0.59 ^b^	31.15 ± 0.62 ^a^	24.11 ± 1.89 ^b^	25.19 ± 0.44 ^b^	0.261	<0.001	0.001
D 21	20.97 ± 0.52 ^b^	26.87 ± 0.47 ^a^	19.62 ± 1.73 ^b^	23.20 ± 1.25 ^ab^	0.082	0.004	0.388
IL-6, pg/mL							
D 1	122.66 ± 1.17	127.79 ± 3.81	132.07 ± 5.57	127.17 ± 6.41	—	—	—
D 15	124.48 ± 2.26 ^b^	157.31 ± 1.55 ^a^	128.58 ± 6.35 ^b^	132.40 ± 1.81 ^b^	0.025	<0.001	0.002
D 21	127.11 ± 1.03	132.48 ± 6.35	126.53 ± 4.23	129.39 ± 1.12	0.871	0.367	0.607
IL-10, pg/mL							
D 1	14.22 ± 0.32	13.84 ± 0.28	13.55 ± 0.14	14.70 ± 0.50	—	—	—
D 15	14.08 ± 0.34 ^b^	10.28 ± 0.22 ^c^	16.32 ± 0.33 ^a^	17.77 ± 0.27 ^a^	<0.001	0.002	<0.001
D 21	14.40 ± 0.20 ^a^	12.39 ± 0.43 ^c^	13.07 ± 0.20 ^bc^	13.90 ± 0.10 ^ab^	0.729	0.040	<0.001
TNF-α, pg/mL							
D 1	42.66 ± 1.81	39.70 ± 1.89	43.83 ± 1.93	37.97 ± 3.33	—	—	—
D 15	42.39 ± 1.13 ^b^	56.12 ± 1.69 ^a^	40.56 ± 3.18 ^b^	46.27 ± 0.91 ^b^	0.004	0.001	0.021
D 21	41.80 ± 0.78	43.98 ± 1.57	42.19 ± 1.30	43.47 ± 0.55	0.963	0.173	0.732

**Table 7:** Relative abundance of dominant rumen bacterial taxa in calves receiving OEO supplementation and/or diquat challenge.

Index	CON	DIQ	OEO	MIX	*p*-Value
Phylum					
Bacteroidota	37.45 ± 29.42	22.13 ± 19.61	59.42 ± 15.34	31.10 ± 21.25	0.06
Firmicutes	30.09 ± 15.50	36.80 ± 9.90	31.03 ± 12.32	29.25 ± 12.99	0.62
Actinobacteriota	27.94 ± 29.21	29.60 ± 19.91	4.04 ± 6.14	31.68 ± 21.21	0.06
Patescibacteria	1.76 ± 1.56 ^b^	9.60 ± 9.89 ^a^	0.82 ± 0.49 ^b^	2.96 ± 1.79 ^b^	0.03
Proteobacteria	2.06 ± 1.47	1.19 ± 0.50	3.92 ± 2.40	4.51 ± 3.91	0.08
Genus					
Prevotella	33.47 ± 28.03	19.65 ± 19.34	55.26 ± 16.13	27.66 ± 20.73	0.09
Olsenella	27.75 ± 29.11	29.33 ± 19.85	3.89 ± 6.11	31.48 ± 21.11	0.06
Lachnospiraceae_NK3A20_group	5.34 ± 5.32	5.36 ± 2.49	1.26 ± 1.57	4.16 ± 3.10	0.07
Ruminococcus_gauvreauii_group	5.06 ± 5.27	6.84 ± 5.23	1.61 ± 2.65	2.60 ± 2.01	0.14
norank_o__Saccharimonadales	1.75 ± 1.56 ^b^	9.60 ± 9.89 ^a^	0.81 ± 0.49 ^b^	2.95 ± 1.79 ^b^	0.03
Syntrophococcus	2.36 ± 1.82	3.95 ± 1.70	2.26 ± 2.22	5.92 ± 7.61	0.32
norank_o__Clostridia_UCG-014	2.66 ± 2.22	5.27 ± 3.53	1.65 ± 1.86	2.98 ± 2.35	0.14
Dialister	2.11 ± 2.06	3.00 ± 3.83	4.49 ± 4.39	1.92 ± 1.64	0.46
Succiniclasticum	2.23 ± 2.05	1.46 ± 1.3	4.90 ± 3.69	2.71 ± 2.04	0.08
Succinivibrionaceae_UCG-001	1.22 ± 1.52	0.38 ± 0.15	3.03 ± 2.31	2.66 ± 3.78	0.13

Note: Data are presented as mean ± SEM (*n* = 6 per group). Different superscript letters within the same row indicate significant differences among groups (*p* < 0.05).

## Data Availability

The dataset presented in this study can be found under accession number PRJNA1354802 in the NCBI Sequence Read Archive (SRA).

## Abbreviations

CON	control group
DIQ	diquat group
OEO	oregano essential oil
MIX	oregano essential oil plus diquat group
ROS	reactive oxygen species
Nrf2	nuclear factor erythroid 2-related factor 2
ADG	average daily gain
DMI	dry matter intake
BW	body weight
16S rRNA	16S ribosomal RNA
MDA	malondialdehyde
8-iso-PGF2α	8-iso-prostaglandin F2α
SOD	superoxide dismutase
GSH-Px	glutathione peroxidase
CAT	catalase
T-AOC	total antioxidant capacity
AST	aspartate aminotransferase
ALT	alanine aminotransferase
ALP	alkaline phosphatase
IL-1β	interleukin-1β
IL-6	interleukin-6
IL-10	interleukin-10
TNF-α	tumor necrosis factor-α
HMOX1	heme oxygenase 1
GCLC	glutamate-cysteine ligase catalytic subunit
GCLM	glutamate-cysteine ligase modifier subunit
SOD1	superoxide dismutase 1
ARE	antioxidant response element
GPX4	glutathione peroxidase 4
NQO1	NAD(P)H quinone oxidoreductase 1
Keap1	Kelch-like ECH-associated protein 1
NF-κB	nuclear factor kappa B
TMR	total mixed ration
qPCR	real-time quantitative polymerase chain reaction
OTU	operational taxonomic unit
PCoA	principal coordinate analysis
PLS-DA	partial least squares discriminant analysis
OPLS-DA	orthogonal partial least squares discriminant analysis
KEGG	Kyoto Encyclopedia of Genes and Genomes
AGE-RAGE	advanced glycation end products–receptor for advanced glycation end products
MAPK	mitogen-activated protein kinase
SCFAs	short-chain fatty acids
RCBD	randomized complete block design
ANCOVA	analysis of covariance
LSMeans	least-squares means
KR	Kenward–Roger
Q–Q plot	quantile–quantile plot
